# Design and synthesis of cyclic lipidated peptides derived from the C-terminus of Cx43 for hemichannel inhibition and cardiac endothelium targeting†

**DOI:** 10.1039/d4md00850b

**Published:** 2024-12-21

**Authors:** Debora Iaculli, Jade Montgomery, Arthur Lamouroux, Anne Caufriez, Rafael Gozalbes, Mathieu Vinken, Filippo Molica, Brenda R. Kwak, Steven Ballet

**Affiliations:** a Research Group of Organic Chemistry, Departments of Bioengineering Sciences and Chemistry, Vrije Universiteit Brussel Brussels Belgium steven.ballet@vub.be; b Department of Pathology and Immunology, Faculty of Medicine, University of Geneva Geneva Switzerland; c Geneva Center for Inflammation Research, Faculty of Medicine, University of Geneva Geneva Switzerland; d Department of Pharmaceutical and Pharmacological Sciences, Vrije Universiteit Brussel Brussels Belgium; e ProtoQSAR SL, Parque Tecnológico de Valencia Paterna Valencia Spain

## Abstract

A peptide segment that is 10 residues long at the C-terminal (CT) region of Cx43 is known to be involved in interactions, both with the Cx43 protein itself and with other proteins, that result in hemichannel (HC) activity regulation. Previously reported mimetic peptides based on this region (*e.g.*, **αCT1**, **CT10**) have been revealed to be promising therapeutic agents in the context of cardiovascular diseases. In this work, novel approaches, such as C- and N-terminal modification and cyclization, to improve the proteolytic stability and bioavailability of the **CT10** peptide are presented. These efforts resulted in a set of unprecedented potent cyclic inhibitors of HC-mediated ATP release with a half-life largely exceeding 24 hours. Additionally, the introduction of a lipophilic moiety with different solubilizing linkers led to the generation of a novel series of water-soluble and lipidated peptides that exhibited high inhibitory capacity in *in vitro* assays at submicromolar concentrations. A cardiac endothelium targeting strategy was also adopted, exploiting the ability of the CRPPR peptide to selectively deliver the peptides to endothelial cells.

## Abstract 2

Potent and stable cyclic peptide inhibitors of Cx43 were identified. Their therapeutic potential was enhanced by the addition of a lipid motif (preserving water solubility), and a targeting peptide, for delivery to cardiac endothelial cells.

## Graphical abstract

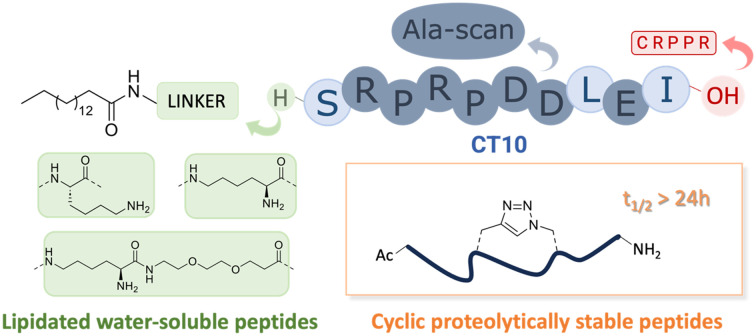

## Introduction

**Fig. 1:**
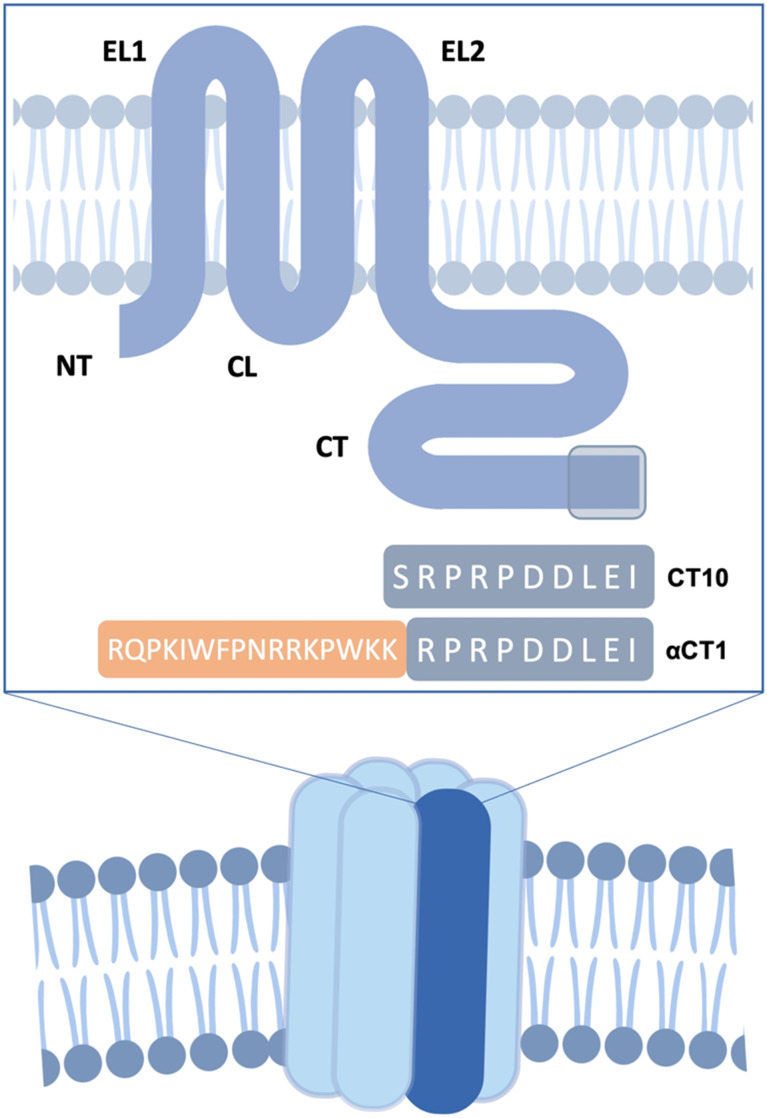
Structure of a hexameric Cx43 hemichannel. EC: extracellular domain. IC: intracellular domain. A single Cx43 monomer is highlighted in dark blue. The transmembrane Cx43 protein is represented in the box. EL1 and EL2: extracellular loops. CL: cytoplasmic loop. NT and CT: cytoplasmic N- and C-terminal tails. The reference sequences of **CT10** and **αCT1** are shown, with the mimicked region of the CT highlighted in grey.

Connexins are membrane-bound proteins that oligomerize into hexameric hemichannels (HCs), which, in turn, form intercellular channels, known as gap junction (GJ) channels.^1,2^ The topology of connexins consists of four transmembrane helices, two extracellular loops, one intracellular loop and cytoplasmic N- and C-termini. The most abundantly expressed member of the connexin protein family is connexin43 (Cx43; Fig. 1).^3^ This connexin is well known for its expression in cardiomyocytes and its crucial role in action potential conduction, but it can also be found in non-cardiomyocyte populations of the heart, such as endothelial cells, smooth muscle cells, fibroblasts and resident macrophages.^4^ Under resting conditions, GJ channels are predominantly in an open state enabling direct cell-to-cell communication, while HCs are principally closed but open up in response to pathological stimuli.^5^ These channels are permeated by ions and signalling molecules (most notably adenosine triphosphate (ATP)) up to 1 kDa in mass.^6,7^ Cx43 HC activity has been linked to numerous cardiac diseases and pathologic mechanisms such as myocardial infarction,^8^ ischemia–reperfusion injury,^9^ and heart failure.^10^ HCs open up in response to ischemic insults, resulting in the release of pro-inflammatory molecules, such as ATP and other essential metabolites from the cells, as well as leading to excessive entry of Na^+^ and Ca^2+^.^11,12^ Therefore, selective inhibition of Cx43 HC activity offers a potential therapeutic intervention in the treatment of cardiovascular diseases.^13^

Different types of small-molecule compounds are known to block connexin HCs, such as glycyrrhetinic acid and its derivatives, carbenoxolone (**Cbx**), long chain alcohols, as well as biologics, such as RNA- and antibody-based inhibitors.^2^ However, small-molecule compounds do not always distinguish between gap junctional and hemichannel activity as well as between different connexin subtypes and may have effects on other cellular targets.^14^ Furthermore, peptide derivatives combine high selectivity with ease of synthesis. A handful of peptides that mimic short portions of Cx43's extracellular or intracellular domains have been developed over the past few decades and have displayed encouraging therapeutic results both *in vitro* and *in vivo*.^15–21^ In particular, **αCT1** (H-RQPKIWFPNRRKPWKKRPRPDDLEI-OH; Fig. 1), a peptide developed by the Gourdie group^22^ mimicking the last 9 amino acids of the Cx43 C-terminal region (CT), which complemented to the antennapedia cell-penetrating peptide penetratin, has been shown to be a promising therapeutic agent preserving left ventricular (LV) function after cardiac I/R injury.^22,23^ It has undergone multiple clinical trials in cutaneous injuries in humans and is currently in phase 3 of development for cutaneous radiation injury.^24^

A nonapeptide comprising the same sequence but devoid of the cell-penetrating motif, named **αCT11** (H-RPRPDDLEI-OH, not shown), has been shown to be able to preserve LV function when administered as post-ischemic treatment in isolated mouse hearts.^23,25^ Both peptides were originally designed to disrupt the interaction between the Cx43 C-terminal tail and the actin-binding protein zonula occludens-1 (ZO-1),^22,26^ consequently affecting Cx43 trafficking, insertion of HCs into GJs and regulation of HC and GJ channel activity.^26–28^ However, it has been shown that the cardioprotective properties of the above-mentioned peptides are mostly linked to their ability to interact with an α-helical region of the Cx43 CT, called H2 (*i.e.* the protein stretch Asp340–Ala348 (ref. 1)). There are also indications that the peptides interfere with an inter- or intra-molecular interaction between the CT of Cx43 and the cytoplasmic loop (CL) involved in Cx43 HC regulation, as a similar peptide copying the last 10 amino acids of the Cx43 CT linked to a TAT cell-penetrating sequence, **TAT-CT10**, was shown to be able to bind to the CL of the protein.^29,30^ In general, such protein–protein interactions (PPIs) or domain–domain interactions are difficult to address with small-molecule therapeutics, since PPIs are often characterized by a large contact area, which cannot be covered by a small molecule.^31^ Peptide therapeutics, however, are able to interfere with PPIs in a highly selective fashion. As the binding between proteins or domains within these proteins is driven by interactions at crucial amino acid “hot spots”, often associated with the presence of secondary structure motifs, tailor-made peptides that reproduce these elements can bind with high selectivity and affinity to protein targets.^32^ On the downside, however, the rapid proteolytic cleavage of peptide-based therapeutics, together with their low bioavailability, makes them poor systemically applied drug candidates.^33^ In order to enhance their structural stability and resistance to hydrolysis, while maintaining high selectivity and minimal toxicity, peptide macrocyclization is commonly utilized. Macrocyclic peptides present advantages such as a more limited conformational flexibility, which can result in a higher binding affinity, and enhanced protease resistance.^34^ Furthermore, cyclization caused an increase in hydrophobicity, which can result in enhanced bioavailability, leading in some cases to even orally bioavailable compounds.^35^ All these characteristics are allowing cyclic peptides to become increasingly more relevant as therapeutic agents, with more than 40 cyclic peptides being currently used as drugs.^36^

To further increase the therapeutic potential of a drug candidate, targeted delivery to the organ or tissue of interest after systemic treatment is highly desirable. The use of homing peptides, *i.e.* peptides that selectively bind to markers expressed on the surface of different cell types, can increase the specificity and efficacy of drug delivery, while reducing the side effects linked to off-target interactions. A wide variety of peptides homing to different tissues and pathological conditions has been identified, mostly through phage-display techniques, and used as delivery vehicles for drug molecules, oligonucleotides, imaging agents and liposomes.^37^ Notably, the CRPPR pentapeptide has been shown to target the heart with >300 fold selectivity, binding to markers expressed on the cell surface of the cardiac endothelium.^38^ This peptide has been used to target peptides, liposomes and stem cells to the heart vasculature both *ex vivo* and *in vivo*.^39,40^

Here, we present the design rationale, synthesis, and *in vitro* biological evaluation of a set of conformationally stabilized and proteolytically stable peptide inhibitors based on the ten-amino acid-long CT region of Cx43 (*i.e.* the sequence SRPRPDDLEI). The importance of the positively and negatively charged residues in the sequence, as well as the role of proline residues, was assessed by sequentially replacing each of these amino acids with alanine (*i.e.* performing an Ala-scan). Additionally, selected side chain substitutions were introduced in the peptide sequence to allow macrocyclizations through the Huisgen cycloaddition reaction. The peptides were obtained as N-terminal acetylated and C-terminal carboxamides, in order to increase resistance to degradation by aminopeptidases and carboxypeptidases, respectively.^41^ Furthermore, a set of lipopeptides were designed to potentially exploit the ‘pepducin’ approach.^42,43^ Reportedly, the use of lipidic motifs allows for an interaction with the cell membrane, which potentially results in a translocation of the peptide to the intracellular side of the membrane and an anchorage of the active sequence in the vicinity of its target.^44^ The lipid tail was linked to the peptide through cationic linkers of different lengths, in view of improving the water solubility of the analogues and determining the ideal distance between the peptide pharmacophore and the lipid anchor to the cell membrane. The synthesized peptides were tested for their inhibitory activity on Cx43 HCs by *in vitro* ATP release studies, and their proteolytic stability in human plasma was tested. As the obtained peptidomimetics are specifically relevant in the context of treatment of cardiac diseases, and due to the critical role that cardiac endothelial cells play in the initiation and progression of inflammation,^45^ a selective targeting strategy was additionally investigated through the use of the cardiac endothelium-homing peptide CRPPR. This strategy was combined with the pepducin approach, in order to elucidate the impact of these structural changes on cell distribution of the bioactive cargo.

## Results and discussion

### Ala-scan and design of cyclic peptides

**Fig. 2:**
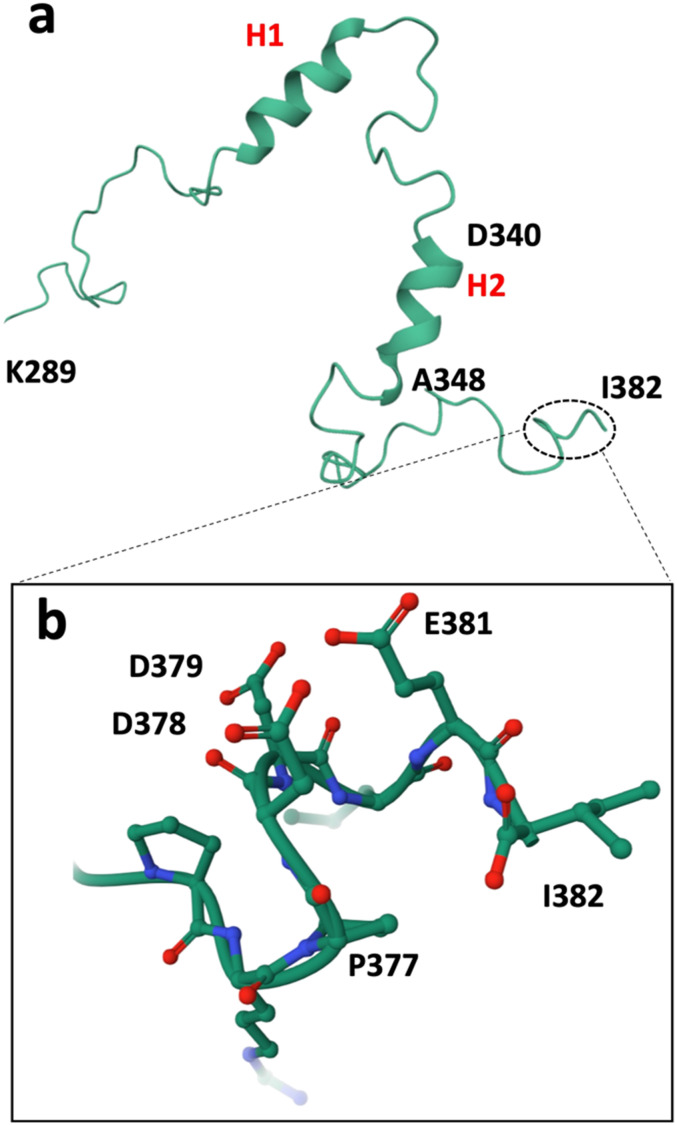
a) Structure of the CT domain of Cx43, based on earlier NMR studies (PDB1R5S).^1^ The α-helical regions H1 and H2 are indicated in red. b) Zoomed image of the C-terminal extremity. The residues are numbered according to their relative positions in Cx43.

The last 10 amino acids of the Cx43 CT were demonstrated to interact with the L2 region (amino acid stretch comprising the residues 119–144) of the CL domain, and this interaction regulates the Cx43 HC activity.^30^ Previous work showed that this “loop-tail interaction” was abolished when two Asp residues (*i.e.* Asp378 and Asp379) were replaced by Ala and when the two Pro residues were replaced by Gly.^30^ Furthermore, **αCT1** was shown to be able to bind to an α-helical region (H2) of the Cx43 CT (Fig. 2a), interfering with a PKC-mediated phosphorylation occurring on the latter.^46^ The three negatively charged residues belonging to the **αCT1** sequence (*i.e.* Asp378, Asp379 and Glu381; Fig. 2b) were shown to be essential for binding with two Lys residues (*i.e.* Lys345 and Lys346) in the H2 region of the Cx43 CT region.^23^

**Fig. 3:**
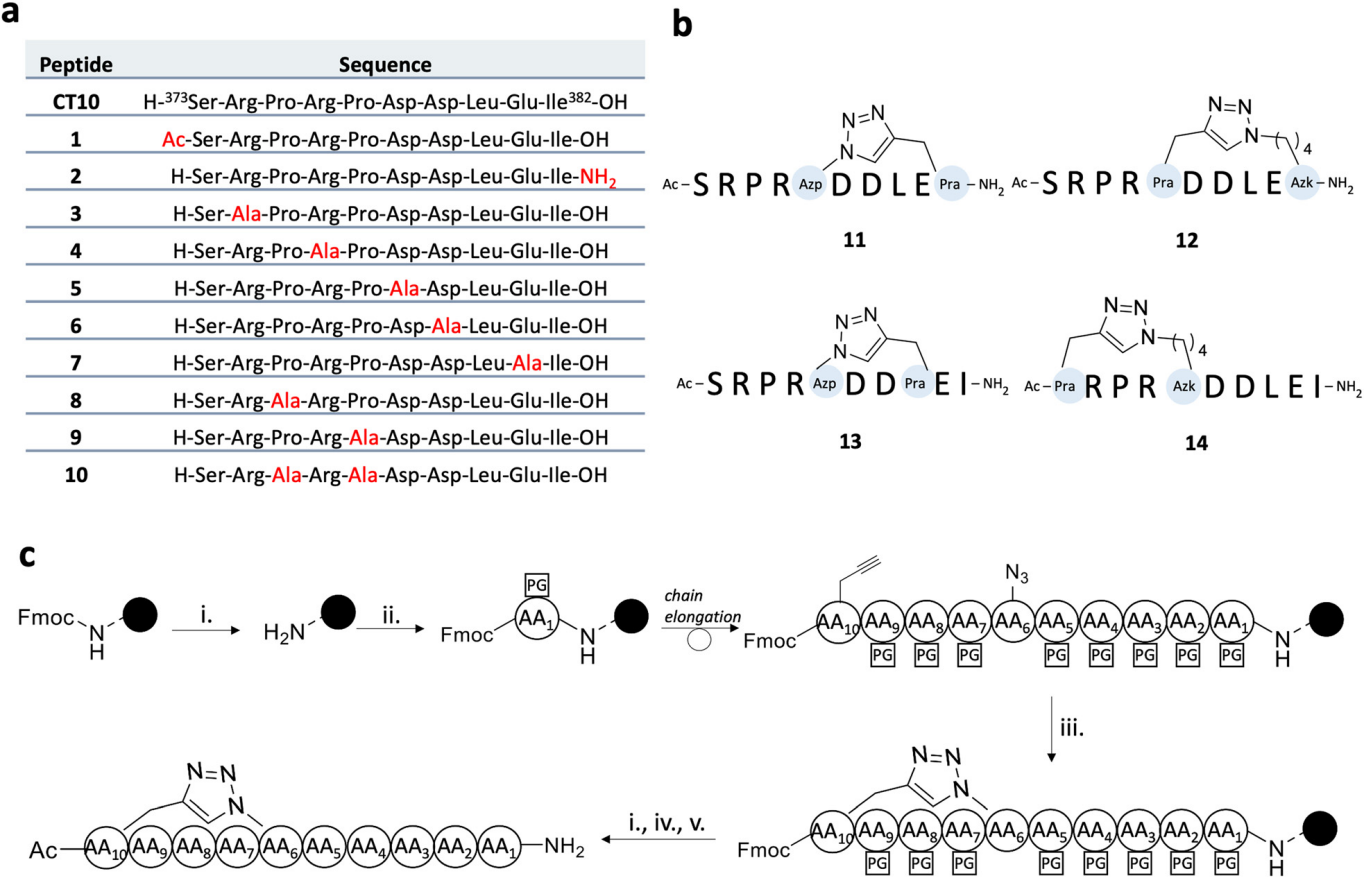
a) Sequences of linear and cyclic **CT10** analogues. The substitutions in the linear sequences are shown in red. b) Cyclic peptides based on the **CT10** sequence. Azp, Pra and Azk stand for (4*S*)-4-azidoproline, propargylglycine and azidolysine, respectively. c) Solid-phase synthesis of cyclic peptides. i. 4-Methylpiperidine 20% in DMF, RT, 1 × 5 min, 1 × 15 min; ii. Fmoc-AA(PG)-OH (1.5 or 3 eq.), HBTU (1.5 or 3 eq.), DIPEA (5 eq.), DMF, RT, 1–2 h; iii. CuBr (3 eq.), Na-ascorbate (3 eq.) in H_2_O, 2,6-lutidine (10 eq.), DIPEA (10 eq.), DMF, RT, 18 h; iv. Ac_2_O (10 eq.), DIPEA (5 eq.), DCM, RT, 45 min; v. TFA : TIPS : H_2_O = 90 : 5 : 5, RT, 3 h.

In the present work, peptides based on **CT10** were designed to further examine the role of individual residues in the inhibitory process, to investigate the importance of the N- and C-peptide termini and to provide analogues displaying a higher proteolytic stability in view of future lead compound optimizations (Fig. 3a). The peptides were synthesized by standard Fmoc/*t*-Bu solid-phase peptide synthesis (SPPS). In peptides 1 and 2, termini were capped *via* acetylation and amidation, respectively, in view of enhanced proteolytic resistance. Then, point mutations to Ala were introduced at the level of the positively charged Arg residues (to give peptides 3 and 4), the negatively charged Asp and Glu residues (peptides 5, 6, and 7) and the Pro residues (peptides 8, 9 and 10).

Additionally, a macrocyclization strategy was investigated. Multiple strategies have been developed to obtain macrocyclic peptides based on side chain-to-side chain cyclization reactions.^47^ Here, the copper-catalysed 1,3-dipolar cycloaddition (CuAAC) reaction was adopted.^48–50^ This “click” reaction has been extensively used in medicinal chemistry, due to its robustness, orthogonality and biocompatibility.^51,52^ As such, four macrocyclic peptides were designed (compounds 11, 12, 13 and 14, Fig. 3b). In all cyclic peptides, the N-terminus was acetylated and the C-terminus was obtained as a carboxamide function. Due to its mostly disordered nature, the C-terminal region of Cx43 could not be structurally determined in the recently published cryo-EM structures of the protein.^53,54^ Nonetheless, the NMR structure of the CT in solution, published by Sorgen and coworkers,^1^ led to the design of a cyclic peptide based on the 10 C-terminal amino acids of the protein, compound 11. This peptide matches the solution structure of the Cx43 CT region (Fig. 2), in which the negatively charged side chains of the Asp and Glu residues are oriented in the same direction, forming an acidic cluster that may play a fundamental role in the binding process.^1^ As such, the Pro377 residue was replaced by a (4*S*)-4-azidoproline (Azp) residue, while the Ile382 residue at the C-terminal extremity was replaced with a propargylglycine (Pra) residue, to allow for the formation of a triazole tether (Fig. 3c). No specific secondary structure stabilization could be obtained for this peptide, as both the linear **CT10** sequence and the cyclic 11 peptides show a random coiled conformation by circular dichroism (CD) spectrometry (Fig. S1†). In peptide 13, the rigid Azp residue already present in 11 was maintained while the length of the covalent tether, interconnecting the residues in positions (*i*, *I* + 5), was varied to (*i*, *I* + 3). Finally, in peptides 12 and 14, the Pro375 residue was replaced by Pra or azidolysine (Azk) residues, respectively, and the triazole ring was moved closer to the N-terminus of the peptide. As the macrocycles are similarly located in almost all peptides and a random coiled conformation was observed for both 11 and **CT10**, no additional CD experiments were conducted.

### Design of lipidated peptides

Peptides tend to have low permeability across cell membranes, which negatively affects their therapeutic efficacy and bioavailability. Different uptake mechanisms can be exploited to increase the delivery of a peptide to cytoplasmic sites, including the use of cell-penetrating peptides (CPPs), *i.e.* peptide sequences that are able to be internalized by cells through different mechanisms, mostly endocytosis.^55,56^ However, the release of the cargo is often limited by endosomal trapping.^57^ An alternative strategy for targeting the intracellular regions of membrane receptors (*e.g.* G protein-coupled receptors, GPCRs) consists of linking the active peptide sequence to a lipidated motif.^42,58^ The lipophilic nature of the latter allows an interaction and anchorage with and within the cell membrane, consequently inducing a “flip” of the peptide to the intracellular site.^44^ These so-called pepducins have been shown to be able to increase the pharmacological activity of peptides mimicking the intracellular domains of GPCRs, by tethering the active sequence in the vicinity of the target receptor and increasing its effective molarity.^59^ Moreover, such constructs have been demonstrated to have functional effects in pathological animal models, as well as showing improved bioavailability, safety and tolerability in human subjects.^58^ Noteworthy, the synthesis of a pepducin is usually drastically less demanding than the synthesis of CPP-conjugated peptides, as the alkyl group (generally derived from palmitic acid) can be easily coupled in one step on the N-terminal end of the resin-bound peptide.

**Fig. 4:**
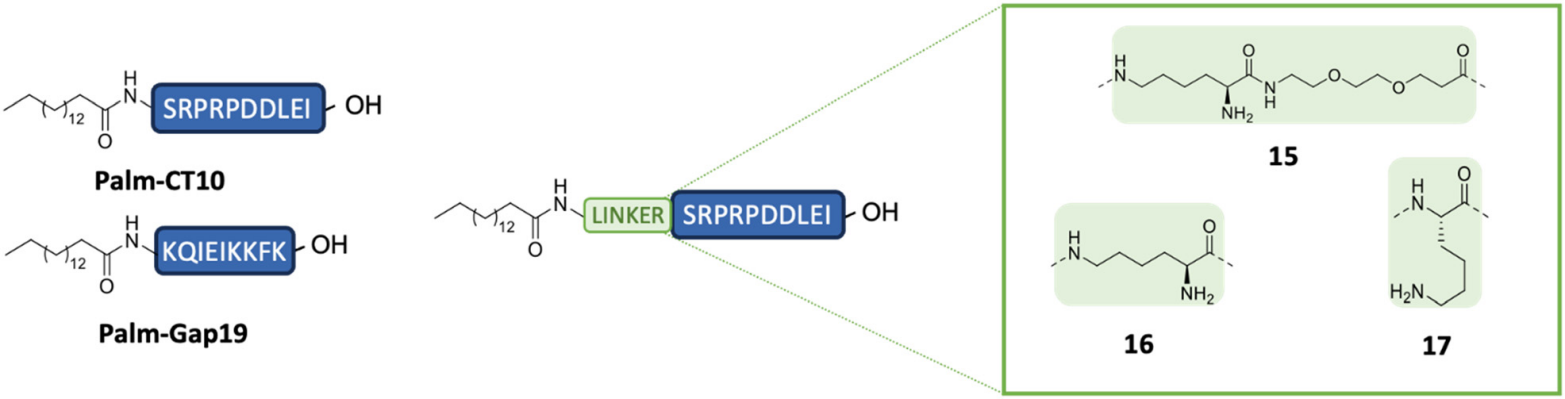
Selected lipidated peptide sequences based on **Gap19** and **CT10** compounds. The different linkers applied are shown in green.

Hence, the long alkyl moiety from palmitic acid was introduced on the N-terminal extremity of **CT10** and on another well-established inhibitor of Cx43 HC activity based on a cytoplasmic loop-derived segment, **Gap19** (H-KQIEIKKFK-OH),^15^ resulting in compounds **Palm-CT10** and **Palm-Gap19**, respectively (Fig. 4). However, as a result of this modification, the overall hydrophobicity of the resulting lipopeptides increased, eventually resulting in a lower water solubility or solubility in buffers which hampered *in vitro* testing. To overcome this limitation, a set of cationic linkers were designed. Three linkers of different lengths and based on a single Lys residue were introduced between the lipid tail and the **CT10** sequence, to investigate how the solubility of the lipopeptide would be affected. In two peptides, palmitic acid was linked to the peptide sequence through a single Lys residue, either in the main chain (Palm-K-SRPRPDDLEI-OH, peptide 17) or *via* the side chain ε-amine (Palm-εK-SRPRPDDLEI-OH, peptide 16). In addition, palmitic acid was also linked to the side chain of a Lys residue which was in turn positioned on a PEG linker (Palm-εK-PEG-SRPRPDDLEI-OH, peptide 15) (Fig. 4). The resulting pepducins were tested *in vitro* for their inhibitory capacity on Cx43 HC-mediated ATP release.

### *In vitro* determination of the inhibitory capacity of the peptides through ATP release studies

**Fig. 5:**
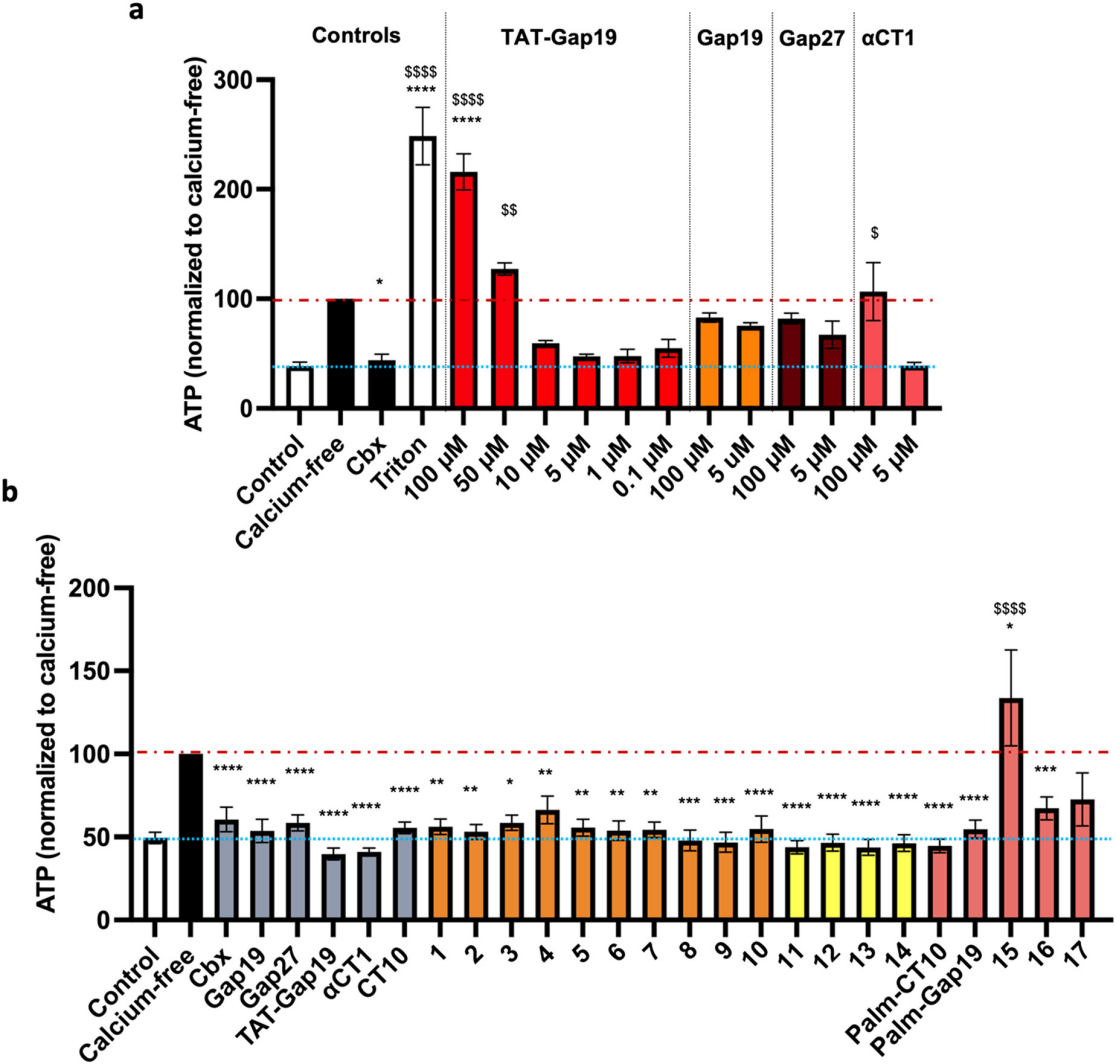
a) Setup of controls for ATP release experiments in B16-BL6 cells. Control represents basal ATP release in HBSS (HC closed condition; blue line), while calcium-free is maximal ATP release through connexin HCs induced by calcium depletion (red line). The nonspecific reference compound carbenoxolone (**Cbx**) was used at 25 μM and the reference peptides **TAT-Gap19**, **Gap19**, **Gap27** and **αCT1** at concentrations ranging from 0.1 μM to 100 μM. Triton X-100 (1%) was used as a cell lysis control. b) ATP release assay in B16-BL6 cells. HCs were opened by calcium depletion (calcium-free, 30 min). **Cbx** was used at 25 μM and all reference peptides and modified analogues were used at 5 μM. The orange bars represent the peptides in which alanine substitutions or C- and/or N-terminal modifications were introduced. The yellow bars represent the cyclic peptides. The red bars represent the lipidated peptides. Data are shown as mean ± SEM ^$^*P* value compared to control conditions, *P value compared to calcium-free conditions. *N* = 3–9.

The inhibitory capacity of the peptides on connexin HC activity was determined *in vitro* by measuring ATP release triggered by a depletion of extracellular Ca^2+^, hereafter called “calcium-free” condition.^60^ The assays were performed on B16-BL6 cells, a murine melanoma cell line endogenously expressing Cx43.^61^ To measure ATP release, B16-BL6 cells were rinsed with a physiological Hanks' balanced salt solution (HBSS; control) followed by incubation for 5 minutes in HBSS containing the peptides. After rinsing with calcium-free HBSS (Ca-free), the cells were incubated with Ca-free containing the peptides for 30 minutes. Positive and negative control conditions included 30 minute incubation with HBSS, Ca-free without peptides or Ca-free containing 25 μM of the non-selective small-molecule inhibitor **Cbx**. ATP release in the supernatant was measured by bioluminescence. In order to determine the optimal concentration of peptides to be used in the assays, an initial round of testing was conducted on the control peptides **αCT1**, **Gap27**,^62^**Gap19** (ref. 15) and **TAT-Gap19** (ref. 15) as well as **Cbx**.^63^ A doubling of ATP release was observed 30 minutes after the switch to the calcium-free solution, indicating effective Cx43 HC channel opening under these conditions (Fig. 5a and Table S1†). As expected, the nonspecific connexin HC inhibitor **Cbx** (25 μM) reduced calcium-free-induced ATP release to nearly control conditions. At a high concentration (100 μM), **Gap19**, **Gap27** and **αCT1** had minimal to no effect on ATP release while **TAT-Gap19** had the opposite effect inducing the release of ATP to a similar extent as the detergent Triton X-100 (1%). This might be due to the presence of the CPP motif, causing some disturbance at the level of the cellular membrane, and correlated with the previous findings on the use of the peptide at high concentrations.^64,65^ Our results on the control peptides **TAT-Gap19** and **Gap19** match earlier observations that showed a higher inhibitory capacity for the TAT-conjugated peptide. The **Gap19** peptide exhibited an IC_50_ of about 50 μM on Cx43-mediated ATP release, while **TAT-Gap19** had an IC_50_ of 7 μM, the same as **Gap19** when applied intracellularly *via* a whole-cell recording pipette, indicating that the peptide is acting intracellularly and that, despite showing some membrane permeation properties, its intracellular delivery is increased by the TAT translocation motif.^15^ In our experiments, **TAT-Gap19** showed a higher inhibitory capacity on ATP release at 0.1 μM concentration than **Gap19** did at 100 μM (Fig. 5a). At 5 μM, all peptides showed more or less inhibitory capacity. As such, subsequent ATP release experiments were performed at a concentration of 5 μM (Fig. 5b and Table S2†).

None of the alanine substitutions in the linear sequence, nor C- and/or N-terminal modifications (shown in orange bars, peptides 1 to 10) led to a significant decrease in inhibitory capacity, as compared to **CT10**. Previously, the Gourdie group reported that replacing the glutamic acid residue in the penultimate position of the **αCT1** sequence leads to a diminished ability of the peptide to interact with Cx43-CT but has no effect on the interaction with the PDZ2 domain of ZO-1.^23^ Our results on peptides 5, 6 and 7, in which each of the negatively charged amino acids was replaced by alanine, seem to indicate that the loss of one negative charge is not sufficient to abrogate the inhibitory activity of the peptide.

**Fig. 6:**
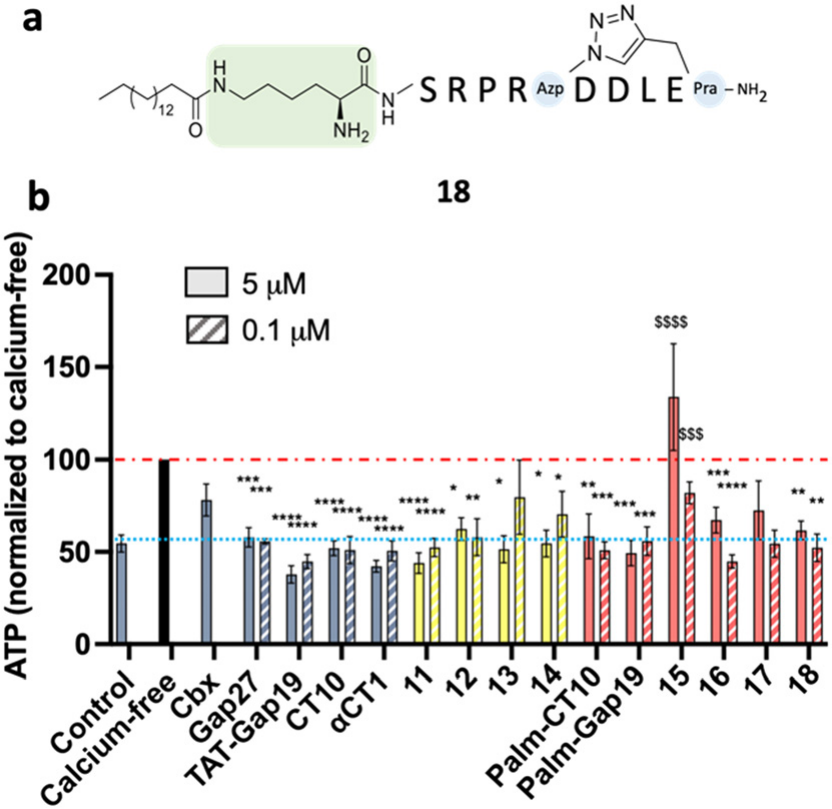
a) Structure of cyclic lipidated peptide 18. b) ATP release assay in B16-BL6 cells. Hemichannels were opened by calcium depletion (calcium-free, 30 min). **Cbx** was used at 25 μM and all reference peptides and modified analogues were used at 5 μM (solid bars) or 0.1 μM (hatched bars). The yellow bars represent the cyclic peptides. The red bars represent the lipidated peptides. Data are shown as mean ± SEM. ^$^*P* value compared to control conditions, *P value compared to calcium-free conditions. *N* = 5–10.

All the cyclic peptides (shown in yellow bars, peptides 11 to 14) were able to inhibit Cx43 HC-mediated ATP release. In peptides 11 and 12, the C-terminal Ile residue was replaced by Pra and Azk, respectively. This aliphatic branched-chain residue has been shown to be essential for the interaction of the peptide, as well as the whole Cx43 protein, with the second PDZ domain of ZO-1.^23,29,66,67^ The retained activity of the Ile-lacking peptides might confirm the previous findings that the interaction with ZO-1 is less involved in HC activity regulation, as compared to intramolecular Cx43 interactions.^23,29^ The cyclic peptides were also tested at a concentration of 0.1 μM (shown in Fig. 6b as hatched bars), in order to identify the ones among them that are able to retain their inhibitory capacity at lower concentrations, a desirable property for future therapeutic applications. All the cyclic peptide inhibitors maintain some degree of bioactivity, but it is worth noting that peptide 11 performs similarly to **αCT1** without requiring the lengthy cell-penetrating motif. Among the cyclic peptides, only 12 retains a similar activity to 11 and **αCT1** at 0.1 μM. Interestingly, the macrocycle is located at the same position in the sequences of these two cyclic analogues. While both peptides would make good candidates for further optimization, our investigation focused only on compound 11, in light of its slightly higher inhibitory capacity at low concentrations (Fig. 6b and Table S3†). Among the lipidated peptides (shown in Fig. 5c as red bars: **Palm-CT10**, **Palm-Gap19** and peptides 15 to 17), the seemingly best performing ones at a concentration of 5 μM are **Palm-CT10** and **Palm-Gap19**, corresponding to the lipidated analogues of **CT10** and **Gap19**, respectively. In particular, **Palm-Gap19**, despite lacking the presence of a TAT cell-penetrating motif, shows an activity comparable to that of **TAT-Gap19** even at low concentrations (0.1 μM, Fig. 6b). However, these peptides showed limited solubility. Indeed, **Palm-Gap19** was found to be soluble only up to 100 μM in ultra-pure water, while **Palm-CT10** could only be dissolved in the presence of 1% DMSO. The soluble variants of the **CT10**-based lipidated analogues displayed different levels of bioactivity. An increased release of ATP was observed for peptide 15 (comprising the longer εK-PEG linker between the active sequence and the palmitoyl moiety), similar to the result obtained with 100 μM **TAT-Gap19** (Fig. 5b). Thus, the length of the polar linker seems to be related to the inhibitory capacity of the obtained lipopeptide. In this case, the best performing linker was found in peptide 16, in which the palmitoyl group is linked through the side chain of a Lys residue (εK; see also Fig. 4). The linker in peptide 16 was also introduced in a cyclic pepducin based on the peptide 11, giving rise to 18 (Fig. 6a). A potent effect across a wide range of low dosages is desirable in a drug used for patient treatment, and can be seen in all of the control peptides as well as several of the novel peptide analogues, including 11 and 18 (Fig. 6b). In compound 18, the presence of the lipid tail might give additional advantages in future *in vivo* applications, as lipidation is a validated technique to improve physiological stability and increase the circulation time of peptides through albumin binding. Altogether, the latter peptide analogue is able to fully inhibit the calcium depletion-induced release of ATP at a very low concentration, bringing Cx43 HC activity to the level of the control condition.

### *In vitro* plasma stability

**Fig. 7:**
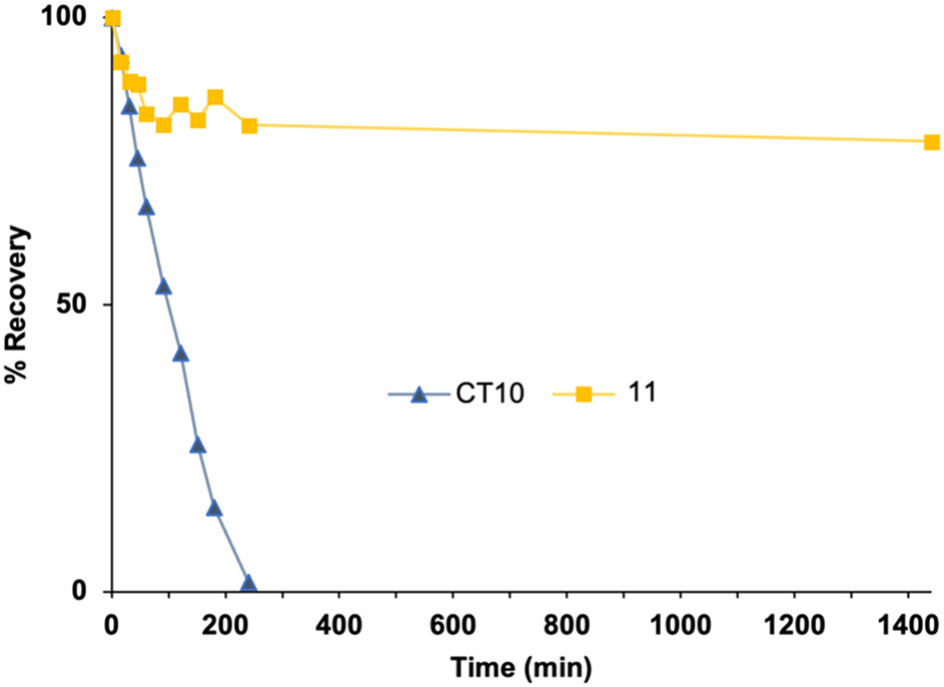
*In vitro* plasma stability of peptides **CT10** and 11. Recovery (%) over time after incubation in human plasma at 37 °C.

To evaluate the proteolytic stabilization brought about by macrocyclization as well as C- and N-terminal amide ‘capping’, *in vitro* plasma stability experiments were carried out on the cyclic analogue **11** and compared to the native sequences **CT10** and on **αCT1**.^68^ For the latter, presumed plasma protein binding was observed, as the peptide precipitated with the proteins when a methanol/TFA solution was added to the samples, despite being soluble in this precipitating mixture, as shown by the fact that the peptide did not precipitate when added to the mixture only after precipitation of the proteins. As such, half-life values could not be determined. For **CT10**, a half-life of 75.86 ± 4.01 minutes was determined (Fig. 7, blue curve). Taking into account the faster degradation commonly observed in serum compared to plasma,^69^ this value correlates with previously reported data on the 9-mer **αCT11** (H-RPRPDDLEI-OH), which was found to be degraded in 30 minutes in mouse serum.^70^ The cyclic analogue 11, however, showed a very high proteolytic stability, with a half-life largely exceeding 24 hours (Fig. 7, yellow curve). The macrocycle introduced in the sequence between the (4*S*)-4-azidoproline residue and the C-terminal propargylglycine residue, together with the amide function on the C-terminus, hence protect the peptide against enzymatic degradation.

### Selectivity assays

**Fig. 8:**
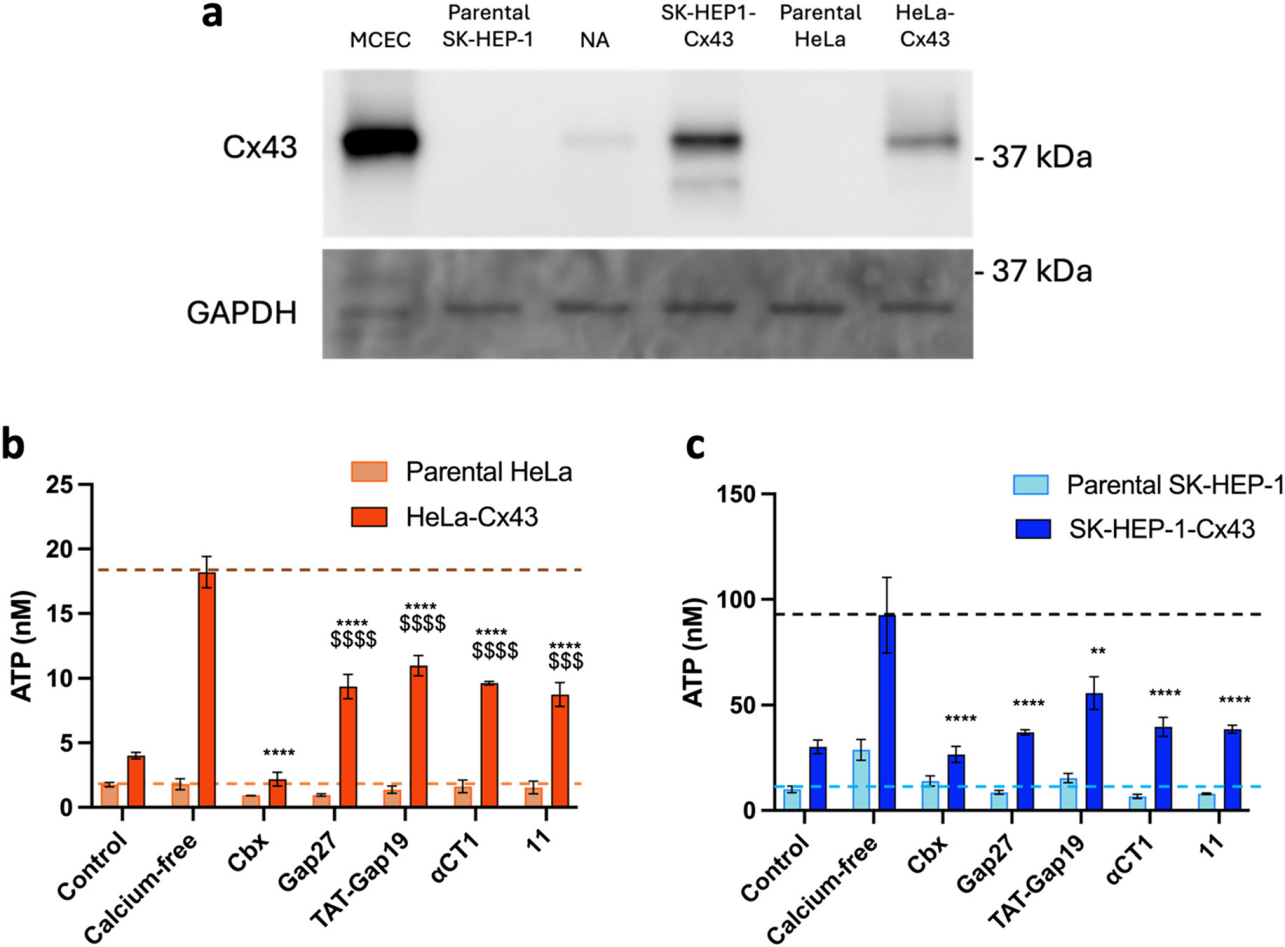
a) Western blot showing the presence or absence of Cx43 in each cell line used for the selectivity experiments. Mouse microvascular cardiac endothelial cells (MCECs) were used as the positive control. GAPDH served as the loading control. NA: non-applicable. b) Communication-incompetent parental HeLa cells lacking functional connexin HCs neither responded to calcium-free conditions nor to any of the Cx43 HC inhibiting compounds, while the HeLa–Cx43 transfectants showed a higher level of ATP release under control conditions, which was 3–4 times increased after calcium removal. **Cbx** (25 μM), control peptides and 10 (all at 5 μM) inhibited the ATP release induced by calcium removal to a varying extent. c) Parental SK-HEP-1 cells, lacking Cx43 but containing Cx45, responded to calcium removal with increased ATP release, which was inhibited by **Cbx** (25 μM), control peptides and 10 (all at 5 μM), showing that the control peptides and 10 are not specific for Cx43 HCs but also affect Cx45 HCs. SK-HEP-1–Cx43 transfectants showed a higher level of ATP release under control conditions, which was 3 times increased after calcium removal. **Cbx** (25 μM), control peptides and 11 (all at 5 μM) inhibited the ATP release induced by calcium removal. Data are shown as mean ± SEM ^$^*P* value compared to control conditions, *P value compared to calcium-free conditions. *N* = 3.

Two cell types were chosen to assess the specificity of the Cx43 analogues. HeLa cells derived from human cervical cancer are the oldest and most commonly used immortalized cells. These cells are so-called communication-incompetent, *i.e.* known to not endogenously express connexins,^71^ while SK-HEP-1 cells, a human liver sinusoid endothelial cell line, are devoid of Cx43 but express a small amount of Cx45.^72^ Together with these parental cells, HeLa cells and SK-HEP-1 cells stably transfected with Cx43 were used (HeLa–Cx43 (ref. 73) and SK-HEP-1–Cx43,^72^ respectively). The presence and absence of Cx43 in the different cell lines were confirmed by Western blotting (Fig. 8a). The specificity of the control peptides **Gap27**, **TAT-Gap19**, **αCT1** as well as the cyclic analogue 11 for Cx43 HCs was assessed through ATP release measurements in these pairs of cells, and is shown in Fig. 8b (and Table S4†) for parental and HeLa–Cx43 cells and in Fig. 8c (and Table S5†) for parental and SK-HEP-1–Cx43 cells. No induction of ATP release was observed when parental HeLa cells were exposed to calcium-free conditions. Moreover, **Cbx**, **Gap27**, **TAT-Gap19**, **αCT1** and 11 did not affect ATP release from these cells. In contrast, ATP release under control conditions was higher in HeLa–Cx43 cells, and calcium-free conditions induced a 4-fold increase in ATP release in HeLa–Cx43 cells, which was completely inhibited by 25 μM **Cbx** and reduced by 40–45% with 5 μM **Gap27**, **TAT-Gap19**, **αCT1** and 11. These results suggest a specific effect of all peptides on Cx43 HC activity in cells devoid of any other connexin. However, a doubling of ATP release was observed when parental SK-HEP-1 cells were exposed to calcium-free conditions, which was attenuated by the treatment with **Cbx** and all peptides, indicating an effect of the compounds on Cx45 HC. Likewise, an inhibitory effect was noticed for all peptides in the SK-HEP-1–Cx43 cells, with **TAT-Gap19** being slightly less effective. These results indicate that while the CT peptides are connexin-specific, they do not seem to be fully selective to the Cx43 isoform. Cx45, despite sharing little sequence homology with Cx43, has been shown to be able to interact with ZO-1 and associate with the Cx43-CT.^74,75^ The inhibitory capacity displayed by both **αCT1** and **11** on SK-HEP-1 cells suggests that either the peptides are able to interact with Cx45 as well or the interaction with ZO-1 can be maintained in cyclic peptides in which Ile has been replaced by a Pra residue. Nonetheless, the cardioprotective properties of **αCT1** are generally ascribed to its interaction with the Cx43 protein,^23^ thus it is reasonable to assume that these properties will be retained by the proteolytically stable 10 peptide.

### Design of cardiac targeting peptides

**Fig. 9:**
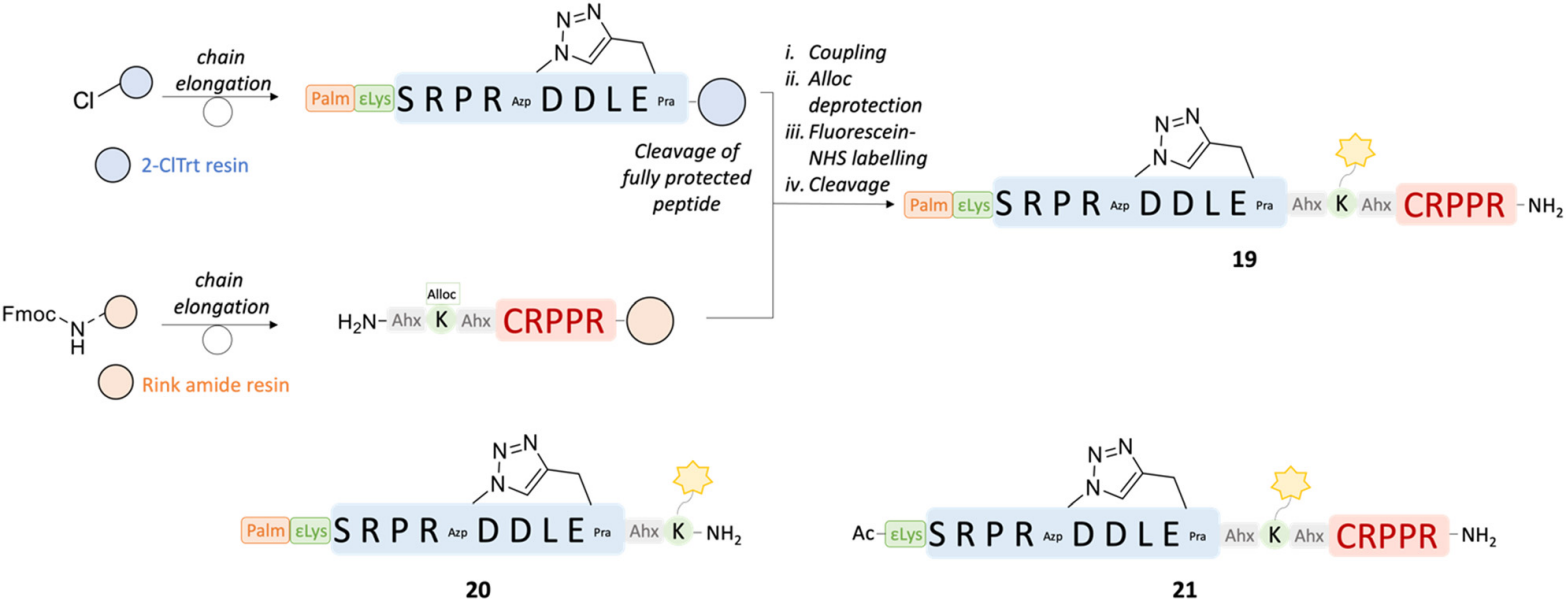
Convergent synthesis strategy adopted for peptides 19 and 21. The sequences of the peptides are shown, together with the sequence of 20, synthesized linearly. The cleavage of the fully protected peptide was performed in 20% HFIP in DCM for 2 h. i) HATU 1.2 eq., DIPEA (2 eq.), DMF, 18 h; ii. PhSiH_3_ (24 eq.), Pd(PPh_3_)_4_ (0.2 eq.), DCM, 2 × 30 min; iii. Fluorescein-NHS (1.05 eq.), DIPEA (2 eq.), DMF, 18 h; iv. TFA : TIPS : H_2_O = 90 : 5 : 5, RT, 30 020 h.

Both **αCT1** and its 9-amino acid-variant **αCT11** demonstrated potent cardioprotective effects to ischemic injury.^23^ Treatment with these peptides could represent the basis for the development of a cardioprotective therapy in the case of acute myocardial infarction in patients with ischemic heart disease. The inflammatory response that follows cardiac I/R injury is complex and involves the activation of multiple cell types, including endothelial cells. Pathological opening of Cx43 HCs in endothelial cells under ischemic conditions is known to contribute to organ damage.^2^ Selective delivery of the Cx43-targeting peptides to the cardiac endothelium could hence provide an additional benefit in the treatment of ischemic heart disease and reduce off-target side effects. In this context, the CRPPR peptide has been shown to improve cardiac endothelium targeting of a cargo by binding to the cell-surface receptor neuropilin-1,^38,76,77^ which is upregulated in endothelial cells after ischemic injury in the peri-infarct region.^78^ Therefore, a set of peptides was designed based on the sequence of 11 in order to investigate the potential of the CRPPR motif for cardiac endothelial cell targeting. The peptides were fluorescently labelled, in order to investigate their binding to MCECs and in cardiomyocyte-like H9c2 cells by flow cytometry experiments. In peptide 19, the CRPPR sequence was added to the C-terminal side of the cyclic lipidated 18 sequence through an Ahx-Lys-Ahx spacer, which also allowed for the addition of the fluorescent tag on the aminoalkyl side chain of the Lys residue (Fig. 9). Two additional fluorescently labelled peptides were synthesized to evaluate the influence on the binding of the homing CRPPR sequence and the lipid tail in the construct, namely compounds 20 (lacking the CRPPR motif) and 21 (lacking the lipid tail). The peptides were synthesized by manual Fmoc-SPPS as described above. Labelling was performed on resin with 5/6-carboxyfluorescein succinimidyl ester (fluorescein-NHS). The homing sequence was added to the C-terminal side of the peptide and the same C-terminus was obtained as a carboxamide, as this terminus was shown to be more effective than its carboxy analogue.^77^ To synthesize peptides 19 and 21, a convergent synthesis strategies was adopted, in which the C-terminal moiety of the peptide, including the homing sequence and the spacer, was synthesized on Rink amide resin, while the N-terminal segment, including the cyclic active peptide sequence with or without the lipid tail, was synthesized on 2-chlorotrityl (2-ClTrt) resin. The 2-ClTrt-resin bound peptide could then be cleaved from the resin, while maintaining all protecting groups intact, and be coupled to the Rink amide-bound sequence.

To test for potential differential preference for various types of heart cells, the binding and/or internalisation of the peptides was investigated by flow cytometry. Murine MCECs and H9c2 cells were incubated with peptides 19, 20 and 21, as well as fluorescein alone, in a physiological (control) buffer for 10 minutes, after which the cells were fixed with 2% paraformaldehyde (PFA) for 5 minutes. The cells were taken up in a fluorescence-activated cell sorting (FACS) buffer and fluorescent cells were analysed by flow cytometry.

**Fig. 10:**
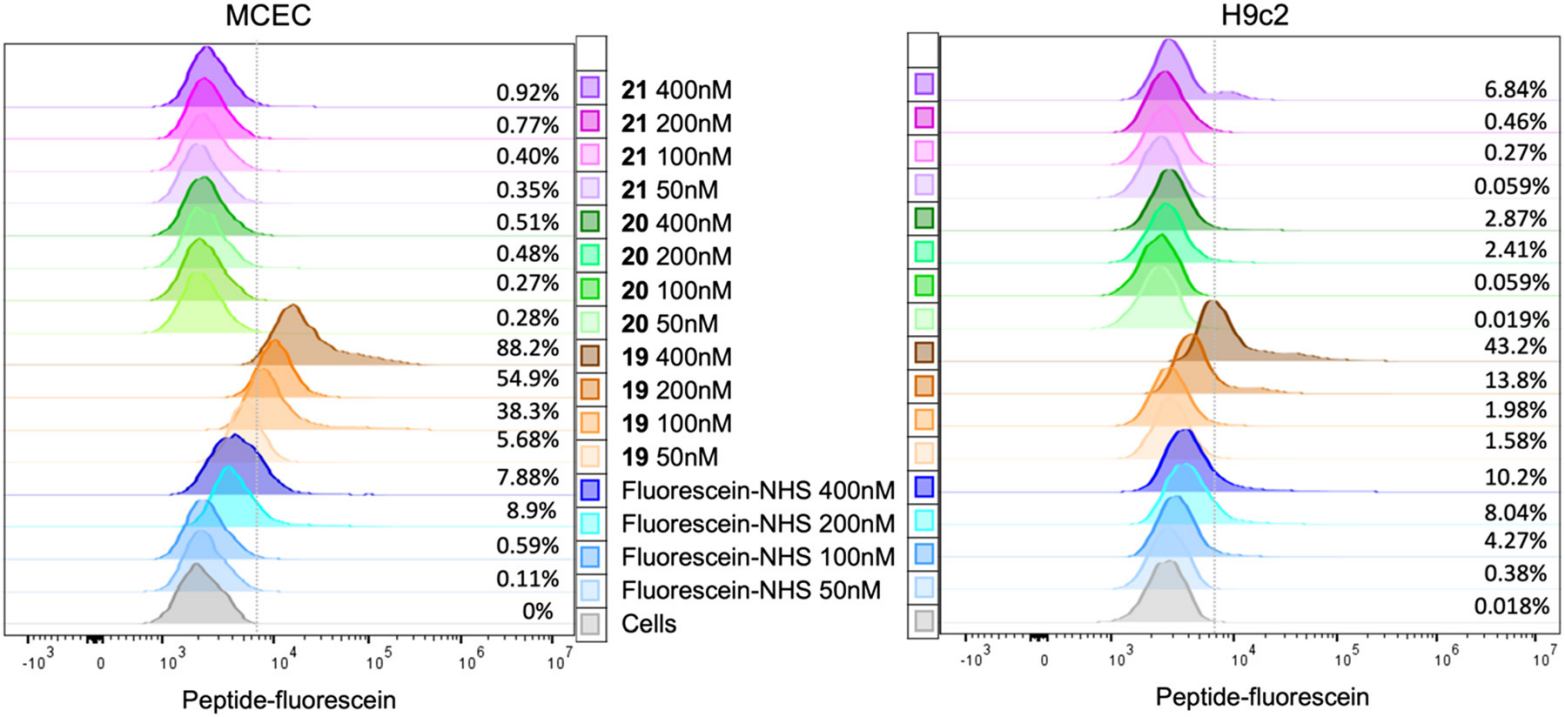
Representative examples of flow cytometry experiments in MCEC and H9c2 cells. The grey curve represents a parallel dish of cells without any staining to measure the level of autofluorescence, which denotes the cut-off value. The percentage noted at the right side of the curves indicates the percentage of fluorescent cells for each condition.

Incubation with peptide 20, comprising the lipid tail but not the targeting sequence, did not result in a fluorescence signal at all concentrations and in both cell types used, indicating that lipidation might not be sufficient to induce cell permeability for peptides comprising a relatively large fluorescein moiety (Fig. 10). Similar results were obtained with peptide 21 (lacking the lipid tail) in all experiments on MCECs and in 2 out of 3 experiments on H9c2 cells. In one experiment on H9c2 cells, a fluorescent signal was obtained when the peptide was used at 200 and 400 μM. Only peptide 19 (containing both the lipidic motif and the cardiac endothelial cell-targeting CRPPR peptide) consistently resulted in a concentration-dependent increase in fluorescence signal in MCECs. Assuming that internalisation is also not possible for this peptide, these results point to a concentration-dependent binding of 19 to cardiac endothelial cells. At the same concentrations, less or no 19 peptide was binding to H9c2 cells, indicating a preference for MCECs.

## Conclusions

This work reports the first cyclic and lipidated inhibitory peptides based on the last 10 amino acids of the Cx43-CT. The cyclic peptide designed based on the NMR structure of the Cx43-CT in solution,^1^11, showed an activity comparable to that of the established peptide inhibitor **αCT1** on Cx43 HC-mediated release of ATP at very low concentrations (5 and 0.1 μM) without requiring the presence of a cell-penetrating motif. While the structural constraint in 11 did not result in secondary structure stabilization and the peptide was shown to be random coiled by CD measurements, the modifications led to a half-life longer than 24 hours in human plasma. *In vitro* biological results obtained on a small library of **CT10**-derived lipidated peptides with different polar linkers allowed for the identification of peptide 16 as the best lipidated **CT10** analogue and revealed the design of the potent water-soluble cyclic lipopeptide 18. The increase in lipophilicity, which is shown by the shift in RP-HPLC retention time (3.01 minutes and 2.89 minutes for 16 and 18, respectively, compared to 1.58 minutes for **CT10** and 1.30 minutes for 11, *cf.* ESI†), might be favourable for *in vivo* bioavailability, and the long half-life of the cyclic peptides might lead to increased efficacy and reduced frequency of administration. While our exploratory work focused on compound 11, the identified optimized lipid tail could be introduced in all macrocyclic analogues reported in this study, as they all showed potent inhibitory capacity and can be expected to possess similar proteolytic stability.

Next to the novel CT-based peptide analogues, our results on the control peptides **TAT-Gap19** and **Gap19** match earlier observations that the presence of a TAT cell-penetrating sequence leads to a higher inhibitory capacity. In this work, the use of a lipidated motif was established as a valid alternative cell-penetrating strategy for **Gap19**. Indeed, this strategy led to peptide **Palm-Gap19**, which shows an activity comparable to that of **TAT-Gap19** even at submicromolar concentrations and it dissolved in water and HBSS buffer without the need for a solubilizing linker.

Flow cytometry experiments on fluorescently labelled cyclic peptides containing either a lipid tail (20) or a cardiac endothelial cell-targeting motif (21), or both (19), indicated that the presence of both moieties is necessary for the peptide to bind to MCECs and H9c2 cells. The difference in binding of peptide 19 suggests that the lipidation and targeting strategies can be combined in the same construct, and that the presence of the endothelial cell-targeting motif induces increased binding to MCECs compared to the cardiomyocyte-like cell line. Future confocal microscopy experiments revealing peptide location could shed light on the mechanism of binding or entry. Altogether, this work makes novel proteolytically stable and rather selective pharmacological tool compounds available to the connexin research community.

## Materials and methods

### Chemicals and reagents

All Fmoc-protected natural amino acids, Rink amide AM and 2-chlorotrityl chloride resins and 5/6-carboxyfluorescein succinimidyl ester (FITC-NHS) were purchased from BLDpharm. The building blocks Fmoc-Pra-OH, Fmoc-Azk-OH, Fmoc-Ahx-OH, preloaded Fmoc-Ile-Wang and Fmoc-Lys(Boc)-Wang resins, *O*-(benzotriazol-1-yl)-*N*,*N*,*N*′,*N*′-tetramethyluronium hexafluorophosphate (HBTU), and Boc-*trans*-4-hydroxy-l-proline methyl ester [Boc-Pro(4R-OH)-OMe] were purchased from Chem-Impex. Copper(i) bromide, trifluoroacetic acid (TFA), triisopropylsilane (TIPS), ethyl cyano(hydroxyimino)acetate (Oxyma), *N*,*N*′-diisopropylcarbodiimide (DIC), Fmoc-8-amino-3,6-dioxaoctanoic acid (Fmoc-NH-PEG-OH), and Fmoc-*N*-hydroxysuccinimide ester (Fmoc-Osu) were obtained from Fluorochem. Sodium ascorbate, 4-methylpiperidine, diisopropylethylamine (DIPEA), pyridine hydrochloride, pyridine, hydrochloric acid (HCl) 37% in H_2_O and 4 M dioxane, sodium nitrite (NaNO_2_), palmitic acid, dichloromethane (DCM), dimethylsulfoxide (DMSO), dimethylformamide (DMF), acetonitrile (ACN), diethylether (Et_2_O), ethyl acetate (EtOAc), and methanol (MeOH) were purchased from Sigma-Aldrich. Methanesulfonyl chloride (MsCl), sulfuric acid (H_2_SO_4_), and lithium hydroxide monohydrate (LiOH·H_2_O) were purchased from ACROS Organics. Magnesium sulfate (MgSO_4_) was obtained from Thermo Fisher Scientific. Sodium azide (NaN_3_) was purchased from Janssen Chimica. The building block Fmoc-Azp-OH was synthesized as described previously.^79^ The Milli-Q water was obtained after purification using a Millipore Simplicity UV system. Analytical reversed-phase HPLC (RP-HPLC) was performed using a VWR-Hitachi Chromaster HPLC with a Chromolith High-Resolution RP-18C column from Merck (150 mm × 4.6 mm, 1.1 μm, 150 Å). The flow rate was 3 mL min^−1^, and ultraviolet (UV) detection occurred at 214 nm. The solvent system used consisted of 0.1% TFA in ultrapure water (A) and 0.1% TFA in acetonitrile (B) with a gradient from 3 to 100% B over a 6-minute runtime. For liquid chromatography–mass spectrometry (LC/MS) analysis, a Waters 600 system combined with a Waters 2487 UV detector at 215 nm was used as the HPLC unit, and an EC 150/2 NUCLEODUR 300-5 C18 column (150 mm × 2.1 mm, 3 μm, 300 Å) was used as the stationary phase. The solvent system used was 0.1% formic acid in water (A) and 0.1% formic acid in acetonitrile (B), with a gradient going from 3 to 100% B over 20 minutes at a flow rate of 0.3 mL per minutes. The MS unit, coupled with the HPLC system, was a Micromass QTOF-microsystem. For high-resolution mass spectroscopy, a UPLC–MS system was used. A Waters Acquity*TM* Premier system equipped with a Waters PDA detector at 215 nm was used as the UPLC unit and an Acquity*TM* Premier BEH C18-column (2.1 mm × 50 mm, 1.7 mm) was used as the stationary phase. The solvent system used was 0.1% formic acid in water (A) and 0.1% formic acid in acetonitrile (B) with a gradient going starting at 1% B for 1 minute followed by a linear gradient to 99% B over 5 minutes at a flow rate of 0.8 ml min^−1^. The MS unit, coupled to the UPLC system, was a Waters Synapt XS QTOF system with an Electron Spray inlet. Lock Mass correction was performed using a Leucine Enkephalin (50 pg μl^−1^) solution in H_2_O : CH_3_CN (1 : 1) with 0.1% formic acid.

### Peptide synthesis

All peptides were synthesized *via* a standard Fmoc-based solid-phase peptide synthesis (Fmoc-SPPS) procedure either manually or using an automated peptide synthesizer (CEM Liberty Lite™). The peptides were synthesized on preloaded Fmoc-Ile-Wang resin (loading 0.57 mmol g^−1^), preloaded Fmoc-Lys(Boc)-Wang resin (loading 0.64 mmol g^−1^) on Rink-amide AM resin (loading 0.4–0.9 mmol g^−1^) depending on the desired C-terminus. Lower loading resin was used for cyclic peptides. For manual SPPS, 3 eq. of Fmoc-protected amino acids (1.5 eq. for unnatural amino acids) were used for each coupling together with HBTU (3 eq. for natural amino acids and 1.5 eq. for unnatural amino acids) and DIPEA (5 eq.) in DMF, for 45–120 minutes. In the case of arginine couplings, double coupling was performed. For automated synthesis, the coupling was performed in the presence of 1 M Oxyma and 0.5 M DIC in DMF, with 5 eq. of Fmoc-protected amino acid. Fmoc deprotection was achieved by treating the resin with a 20% solution of 4-methylpiperidine in DMF, for 5 and 15 minutes. Acetylation was performed with acetic anhydride (Ac_2_O, 10 eq.) and DIPEA (5 eq.) in DCM.

For the cyclic peptides, cyclization was achieved on resin by copper-catalyzed azide–alkyne cycloaddition (CuAAC). After swelling, degassed DMF was added to the resin with CuBr (3 eq.) and 0.5 M aqueous sodium ascorbate (3 eq.). Subsequently, 2,6-lutidine (10 eq.) and DIPEA (10 eq.) were added. The solution was flushed with argon for 5 minutes and shaken for 18 hours. The resin was then washed with DMF (×3), 1 M pyridine hydrochloride in DCM/MeOH = 95/5 (until the resin returned yellow), MeOH and DCM (×3).

For the lipidated peptides, palmitic acid coupling was performed by treating the peptide with palmitic acid (5 eq.), HATU (5 eq.) and DIPEA (10 eq.) in DCM for 2 h. For the analogues in which palmitic acid is coupled to the side chain of lysine, Fmoc-Lys(Alloc)-OH was used. Alloc deprotection was performed on resin on the Fmoc-protected peptide with PhSiH_3_ (24 eq.), Pd(PPh_3_)_4_ (0.2 eq) in DCM for 30 minutes and then repeated. Afterwards, the resin was washed with a 11.6 mM solution of sodium diethyldithiocarbamate and 0.01% (v/v) DIPEA in DMF.

For peptides 19 and 20, a convergent synthesis strategy was adopted. The N-terminal side of the peptide (H-Ahx-Lys-Ahx-Cys-Arg-Pro-Pro-Arg-NH_2_) was synthesized on Rink amide resin (0.05 mmol, loading = 0.412 mmol g^−1^), as previously described using Fmoc-Lys(Alloc)-OH. The remaining moieties of the peptides were synthesized on 2-chlorotrityl resin (0.1 mmol, loading = 0.8 mmol g^−1^) and the linear fully protected peptides were cleaved using 20% HFIP (1,1,1,3,3,3-hexafluoroisopropanol) in DCM for 2 hours. After evaporation of the solvent, the crude peptides were lyophilized and coupled to the resin-bound moieties, using HATU (1.2 eq.) and DIPEA (3 eq.) in DMF overnight. Peptide 21 was synthesized manually directly on Rink amide resin (0.05 mmol, loading = 0.412 mmol g^−1^), as described previously. Afterwards, cyclization and Alloc deprotection were performed as described previously. Fluorescein NHS-ester (FITC-NHS, 1.05 eq.) was linked to the peptides using DIPEA (2 eq.) in DMF overnight.

The cleavage of the peptide from the solid support and simultaneous removal of the side chain protecting groups was performed in a mixture of TFA/TIPS/H_2_O (90/5/5, vol/vol) for 3 hours. After evaporation of the TFA, the peptide was precipitated in cold Et_2_O. The dried solid was dissolved in a H_2_O/ACN (1/1, vol/vol) mixture and lyophilized.

The crude peptides were purified using reverse-phase preparative HPLC (RP-HPLC) on a Gilson HPLC system equipped with Gilson 322 pumps over a Supelco Discovery® BIO Wide Pore C18 column (25 cm × 21.2 mm, 10 μm) using a UV/vis-156 detector at 215 nm, with a solvent system of 0.01% aqueous TFA and 0.01% TFA in ACN. The linear gradients required for purification were adapted depending on the retention time observed on the analytical RP-HPLC. The final products were characterized by analytical RP-HPLC and LC–MS using the systems described above. The purity of the peptides was determined by analytical RP-HPLC and was in all cases above 95%.

### Plasma stability assay

The proteolytic stability of the peptides was determined through *in vitro* stability assays in human plasma as described previously.^68,80,81^ Briefly, a stock solution of the peptide was prepared at a concentration of 2 mM in MilliQ water and was used for further dilutions. A 1120 μM solution was prepared from the stock and incubated with human plasma (1/9 v/v) at 37 °C. Samples were taken at different timepoints and the degradation process was terminated by the addition of cold MeOH + 0.1% TFA. To assess the linearity, accuracy and precision of the method and to determine the half-life of the peptides, 3 independent calibration curves were prepared. The samples were analyzed by RP-HPLC using an Agilent 1200 series gradient HPLC system in combination with an EC HPLC column EC 150/2 NUCLEODUR (C18 HTec, 3 μm length: 150 mm, ID: 2 mm. Macherey-Nagel).

### Circular dichroism

Spectra were recorded using a BIOLOGIC CD spectropolarimeter equipped with a temperature controller using a 1 mm path length quartz cuvette and a scan rate of 5 nm min^−1^. The spectra were recorded in the wavelength range of 190–260 nm with a resolution and a bandwidth of 0.5 and 1.0 nm, respectively. The sensitivity and scan rate of the spectrometer were set to 100 mdeg and 50 nm min^−1^, respectively. The samples were prepared in mQ water to the final concentration of 200 μM. CD spectra were averaged over 3–5 scans with the baseline subtracted from analogous conditions as those for the samples and converted to the mean residue ellipticity.

### Cell culture

Mouse melanoma B16-BL6 cells,^81^ rat H9c2 cardiomyoblasts (ATCC no CRL-1446), mouse cardiac endothelial cells (MCEC; Cedarlane no CLU510), HeLa cells, HeLa–Cx43,^73^ SK-HEP-1 cells and SK-HEP-1–Cx43 (ref. 72) were grown in Dulbecco's modified Eagle's medium (DMEM; Gibco), d-glucose (25 mm), l-glutamine (4 mm) supplemented with fetal bovine serum (FBS, 10%, vol/vol) and penicillin/streptomycin (P/S, 1%, vol/vol) at 37 °C in 5% CO_2_. For MCECs, endothelial cell growth supplement (ECGS, Sigma-Aldrich, 0.75 μg mL^−1^) was added to the above-mentioned medium. B16-BL6 and H9c2 cells were passaged every two days by incubation with trypsin–EDTA (2 minutes at 37 °C) and centrifugation for 5 minutes (290 g) at room temperature. Pellets were resuspended in fresh DMEM. MCECs were passaged every two days by incubation with PBS for 5 minutes at 37 °C, followed by incubation with trypsin–EDTA (2 minutes at 37 °C) and centrifugation for 5 minutes (290 g) at room temperature. Pellets were resuspended in fresh DMEM and plated in culture dishes pre-coated with 1.5% gelatin.

### Western blotting

Western blotting was performed using well-established protocols.^82,83^ In brief, proteins were extracted from cells in RIPA lysis buffer [NP40, 1%; NaCl, 30 mM; Tris, 50 mM (pH 8.0); NaF, 10 mM; Na_3_VO_4_, 2 mM; PMSF, 1 mM; EDTA, 1 mM (pH 7.4); SDS, 0.05%; sodium-deoxycholate, 0.2%; supplemented with a protease inhibitor cocktail]. Protein concentration was measured by a Bicinchoninic Acid assay (BCA; ThermoFisher Scientific) according to the manufacturer's instructions. Cx43 was detected on a PVDC membrane using primary antibodies against Cx43 (1/1000 BD Transduction Laboratories) and glyceraldehyde 3-phosphate dehydrogenase (GAPDH, 1/30 000, Millipore) and appropriate secondary antibodies (1/5000, Jackson Laboratories). Chemiluminescence signals were detected by the Immobilon ECL Ultra Western HRP Substrate (Millipore) using LAS 4000 Fujifilm. GAPDH was used as the loading control.

### ATP release assay

ATP release assays were performed using previously described methods.^60^ Briefly, B16-BL6 cells, HeLa cells or SK-HEP-1 cells were seeded into 96-well plates and grown until they reached confluence. Cells were washed with HBSS at room temperature followed by incubation for 5 minutes in HBSS containing **Cbx** (2.5 μm), Cx43 control peptides or Cx43 analogues at different concentrations ranging from 100 μM to 100 nM. The cells were then washed with calcium-free HBSS and incubated with aforementioned compounds in calcium-free HBSS for 30 minutes. Supernatants were collected and ATP bioluminescence measurements were performed using an ATP bioluminescence kit (Sigma-Aldrich) according to the manufacturer's instructions. ATP release under each condition was expressed as the percentage of calcium-free-induced ATP release. ATP release of cells incubated with HBSS was used as a control for basal ATP release. Triplicates were performed for each independent experiment.

### Flow cytometry

MCECs and H9c2 cells were rinsed 2 times for 5 minutes at room temperature followed by incubation for 10 minutes with peptides 19, 20 and 21, as well as fluorescein alone, in the physiological salt solution at different concentrations ranging from 50 to 400 nM. Thereafter, the cells were fixed with 2% PFA for 5 minutes. Cells were taken up in a FACS buffer [PBS; 0.5% BSA; 500 mm EDTA (pH 7.4)], and fluorescent cells were analysed by flow cytometry.

### Statistical analysis

Statistical analyses were performed using the GraphPad Prism software. One-way or two-way ANOVA followed by Dunnett's test was performed for statistical comparisons. The results are expressed as mean ± SEM. Statistical significance is indicated by *P ≤ 0.05, **P ≤ 0.01, ***P ≤ 0.001 and ****P ≤ 0.0001.

## Author contributions

D. I.: design and synthesis of the peptides, plasma stability experiments, writing – original draft. A. L.: design and synthesis of the peptides, plasma stability experiments, CD experiments, writing – review and editing. J. M.: *in vitro* ATP and selectivity assays, writing – review and editing. F. M.: flow cytometry experiments. Writing – original draft, review and editing. R. G., M. V.: validation, writing – review and editing, funding acquisition. B. R. K., S. B.: validation, writing – review and editing, supervision, funding acquisition.

## Conflicts of interest

There are no conflicts to declare.

## Supplementary Material

MD-016-D4MD00850B-s001

## Data Availability

The data supporting this article have been included as part of the ESI.†
