# Supplementary material for: Design and synthesis of cyclic lipidated peptides derived from the C-terminus of Cx43 for hemichannel inhibition and cardiac endothelium targeting
Source: RSC Med Chem. 2024 Dec 21;16(3):1289–303. doi: 10.1039/d4md00850b (PMC11740094; doi:10.1039/d4md00850b)
Supplement: MD-016-D4MD00850B-s001 [file MD-016-D4MD00850B-s001.pdf]

Supplementary Information (SI) for RSC Medicinal Chemistry.
This journal is © The Royal Society of Chemistry 2024

SUPPORTING INFORMATION

Design and synthesis of cyclic lipidated peptides derived from the *C*-terminus of Cx43 for hemichannel inhibition and cardiac endothelium targeting

Debora Iaculli[a], Jade Montgomery[b,c], Arthur Lamouroux[a], Anne Caufriez[a,d], Rafael Gozalbes[e], Mathieu Vinken[d], Filippo Molica[b,c], Brenda R. Kwak[b,c], Steven Ballet[a*]

[a] Research Group of Organic Chemistry, Departments of Bioengineering Sciences and Chemistry, Vrije Universiteit Brussel, Brussels, Belgium; [b] Department of Pathology and Immunology, and [c] Geneva Center for Inflammation Research, Faculty of Medicine, University of Geneva, Geneva, Switzerland; [d] Department of Pharmaceutical and Pharmacological Sciences, Vrije Universiteit Brussel, Brussels, Belgium; [e] ProtoQSAR SL, Parque Tecnológico de Valencia, Paterna (Valencia), Spain

*Corresponding author. *Email address: steven.ballet@vub.be*

Content

1. **Circular Dichroism (CD) spectra**……………………………………………………2
2. **Biological activity**……………………………………………………2
3. **Peptide characterization**……………………………………………………4
   3a. HPLC chromatograms of peptides……………………………………………………4
   3b. Characterization……………………………………………………15

1. Circular Dichroism (CD) spectra

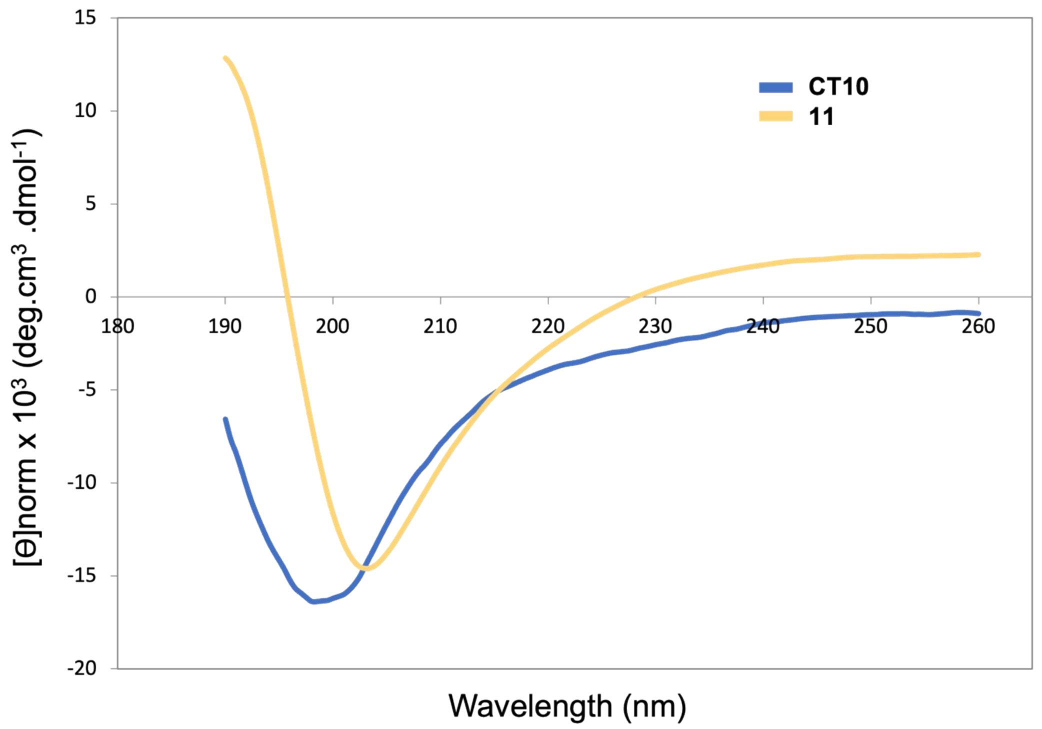

Figure S1. *CD spectra of linear peptide* ***CT10*** *(blue) and cyclic analogue* ***11*** *(yellow) at 200 µM concentration in mQ water. A minimum at 200 nm indicates a random coil conformation.*

2. Biological activity

Table S1*. ATP release data shown in* ***Figure 5a****.*

	ATP release[a]	SEM
**Control**	38.4228	3.8230
**Calcium-free**	100	/
**Cbx**	43.7932	5.5259
**Triton**	248.6069	26.2110
**TAT-Gap19 100 µM**	215.7692	16.4991
**TAT-Gap19 50 µM**	127.3083	5.5652
**TAT-Gap19 10 µM**	59.2634	2.5058
**TAT-Gap19 5 µM**	47.4886	1.8262
**TAT-Gap19 1 µM**	47.6801	6.1592
**TAT-Gap19 0.1 µM**	54.7775	8.0668
**Gap19 100 µM**	82.8115	4.2932
**Gap19 5 µM**	75.4661	2.7612
**Gap27 100 µM**	81.9131	4.8462
**Gap27 5 µM**	67.1248	12.4100
**αCT1 100 µM**	106.6684	26.5628
**αCT1 5 µM**	38.9928	2.9283

[a]*Mean normalized to calcium-free condition.*

Table S2. *ATP release data shown in* ***Figure 5b****. All compounds are used at 5 µM concentration unless otherwise stated.*

	ATP release[a]	SEM
**Control**	49.3877	3.5875
**Calcium-free**	100	/

**Cbx 25 µM**	60.4766	7.7089
**Gap19**	53.6525	6.9376
**Gap27**	58.4393	5.0960
**TAT-Gap19**	39.8281	3.6046
**αCT1**	41.1484	2.3267
**CT10**	55.4854	3.5503
**1**	56.0797	4.7121
**2**	53.0107	4.3760
**3**	58.4702	4.6215
**4**	66.2402	8.3886
**5**	55.5167	5.0309
**6**	53.7892	5.7785
**7**	54.2125	4.6657
**8**	47.8943	6.1749
**9**	46.7801	5.8877
**10**	54.7488	7.8867
**11**	43.9668	4.0310
**12**	46.6075	5.0229
**13**	43.8298	4.8075
**14**	46.2794	4.9171
**Palm-CT10**	44.6669	4.0904
**Palm-Gap19**	54.6035	5.4062
**15**	133.7852	28.8214
**16**	67.2242	7.8381
**17**	72.5860	15.9468

[a]*Mean normalized to calcium-free condition.*

Table S3. *ATP release data shown in* ***Figure 6b****.*

	**ATP release**[a]	**SEM**
**Control**	54.5982	4.6247
**Calcium-free**	100	/
**Cbx 25 µM**	78.1965	8.6963
**Gap27 5 µM**	57.8875	5.2452
**Gap27 0.1 µM**	55.6856	1.3460
**TAT-Gap19 5 µM**	37.8190	4.6963
**TAT-Gap19 0.1 µM**	44.7697	3.7317
**αCT1 5 µM**	42.2267	3.1507
**αCT1 0.1 µM**	50.5858	5.4295
**11 5 µM**	43.9424	5.5982
**11 0.1 µM**	52.4234	4.9713
**12 5 µM**	62.4101	6.1084
**12 0.1 µM**	57.9562	9.9702
**13 5 µM**	51.4148	7.3095
**13 0.1 µM**	79.7386	20.1216
**14 5 µM**	54.5492	7.2228
**14 0.1 µM**	70.4794	12.3845
**Palm-CT10 5 µM**	58.4669	12.1414
**Palm-CT10 0.1 µM**	50.8713	4.5227
**Palm-Gap19 5 µM**	49.4210	6.9054
**Palm-Gap19 0.1 µM**	55.9035	7.7251
**15 5 µM**	133.7852	28.8214
**15 0.1 µM**	81.9773	5.9136
**16 5 µM**	77.0091	12.0120
**16 0.1 µM**	44.8448	3.5623

**17 5 µM**	72.5860	15.9468
**17 0.1 µM**	54.4498	7.2777
**18 5 µM**	61.6970	5.0018
**18 0.1 µM**	52.2265	7.4227

[a]Mean normalized to calcium-free condition.

Table S4. *ATP release data shown in **Figure 8b**. All compounds are used at 5 µM concentration unless otherwise stated.*

	**Parental HeLa**		**HeLa-Cx43**	
	**ATP release[a]**	**SEM**	**ATP release[a]**	**SEM**
**Control**	1.7647	0.1682	4.0067	0.2481
**Calcium-free**	1.7919	0.4219	18.2058	1.2244
**Cbx 25 µM**	0.9207	0.0167	2.1867	0.5339
**Gap27**	0.9529	0.0983	9.3690	0.9358
**TAT-Gap19**	1.3701	0.2847	10.9629	0.7689
**αCT1**	1.6218	0.4958	9.6282	0.1351
**11**	1.5371	0.4916	8.7419	0.9250

[a]Mean (in nM).

Table S5*. ATP release data shown in **Figure 8c**. All compounds are used at 5 µM concentration unless otherwise stated.*

	**Parental SK-HEP-1**		**SK-HEP-1-Cx43**	
	**ATP release[a]**	**SEM**	**ATP release[a]**	**SEM**
**Control**	10.0176	1.5382	30.0127	3.2453
**Calcium-free**	28.7505	4.9305	92.3906	17.9121
**Cbx 25 µM**	13.9931	2.4139	26.4826	3.7740
**Gap27**	8.5784	0.9461	36.9304	1.2387
**TAT-Gap19**	15.3639	2.1802	55.5511	7.7043
**αCT1**	6.6804	1.0241	39.5655	4.5254
**11**	7.9522	0.3945	38.4712	1.9023

[a]Mean (in nM).

3. Peptide characterization

3a. HPLC chromatograms of peptides

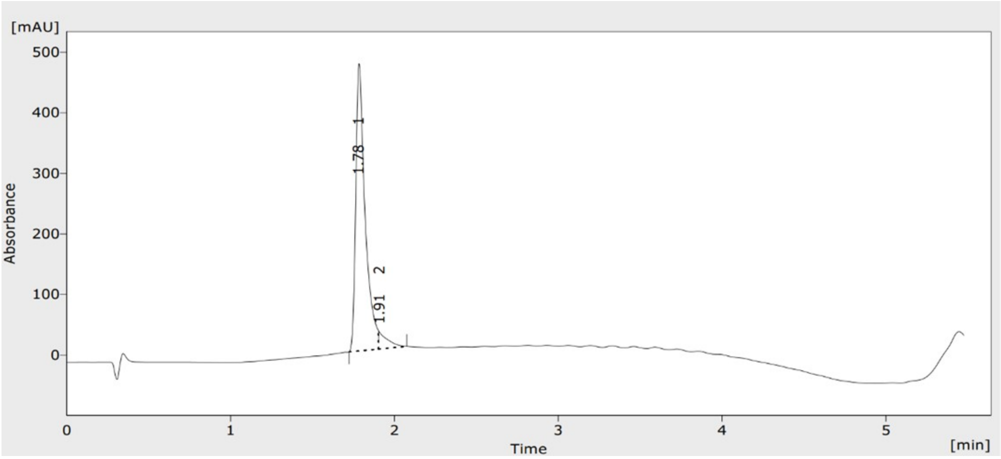

αCT1

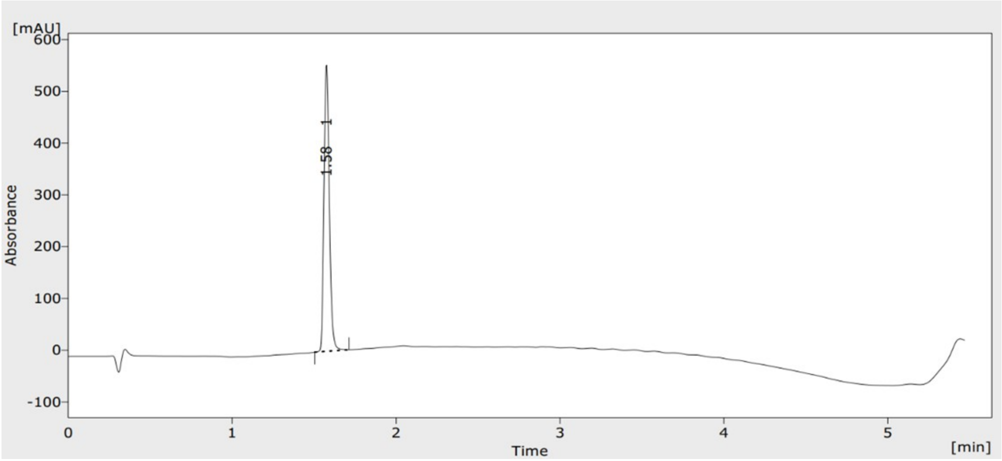

CT10

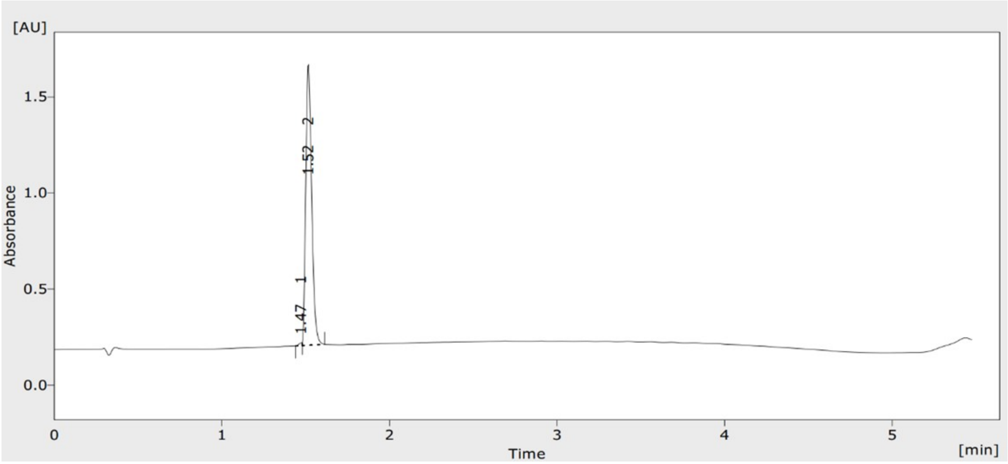

Gap19

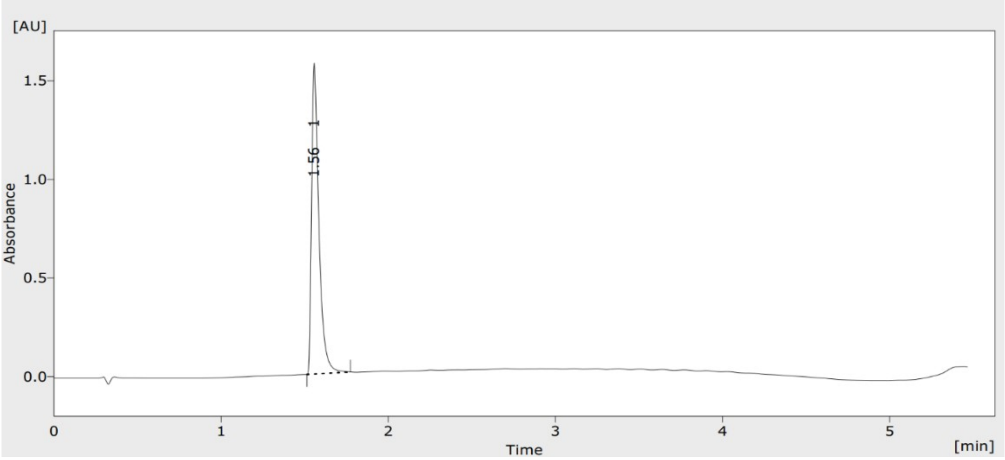

TAT-Gap19

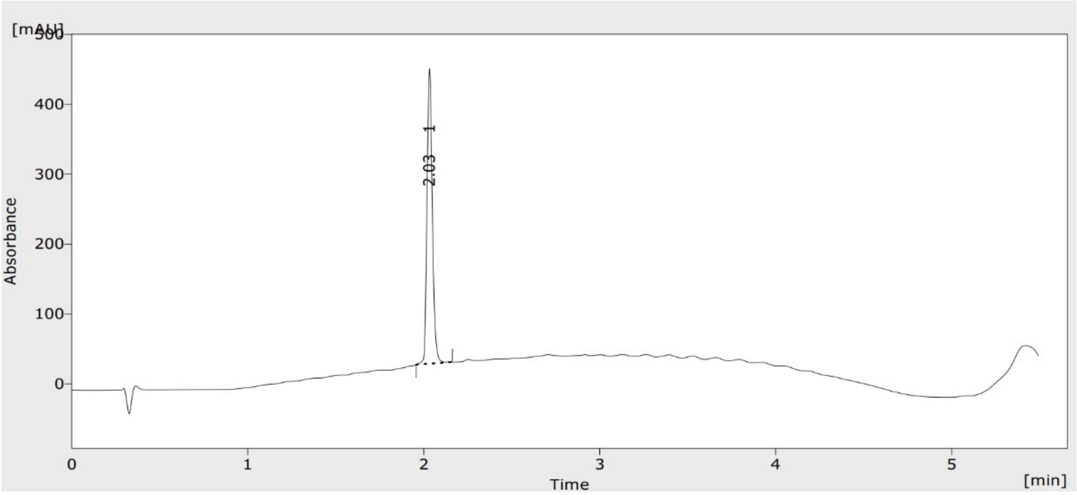

[mAU]
500
400
300
200
100
0
Absorbance
2.03 1
0
1
2
3
4
5
Time
[min]

Gap27

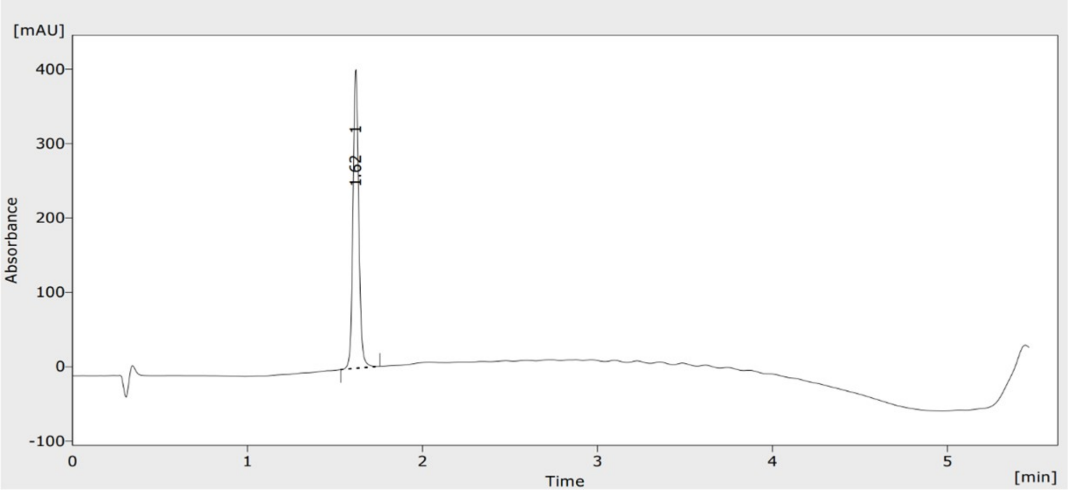

[mAU]
400
300
200
100
0
-100
Absorbance
1.62 1
0
1
2
3
4
5
Time
[min]

1

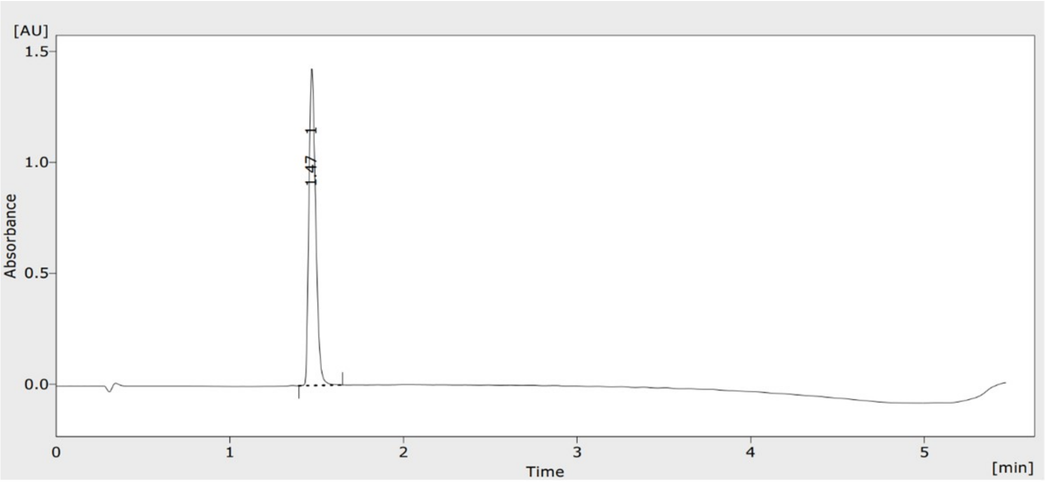

[AU]
1.5
1.0
0.5
0.0
Absorbance
1.47 1
0
1
2
3
4
5
Time
[min]

2

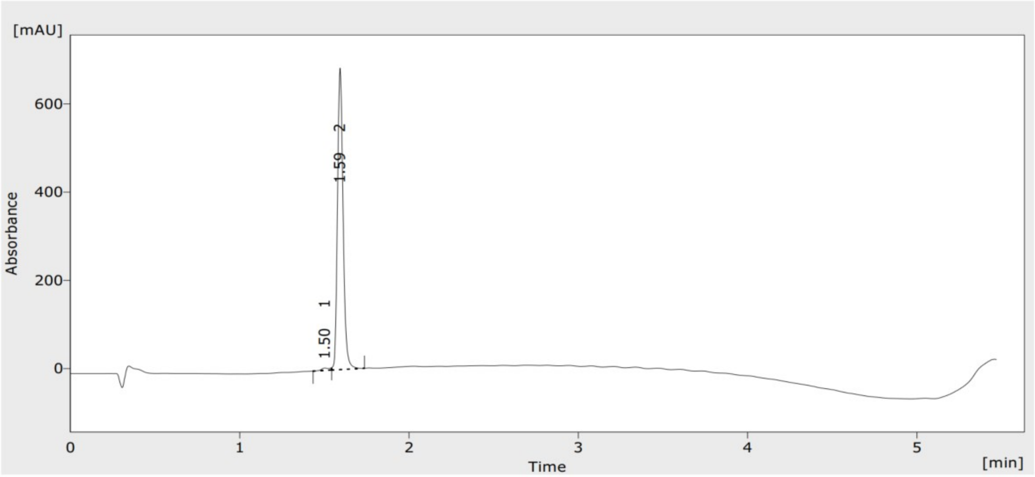
[mAU]
600
400
200
0
Absorbance
1.59 2
1.50 1
0
1
2
3
4
5
Time
[min]

3

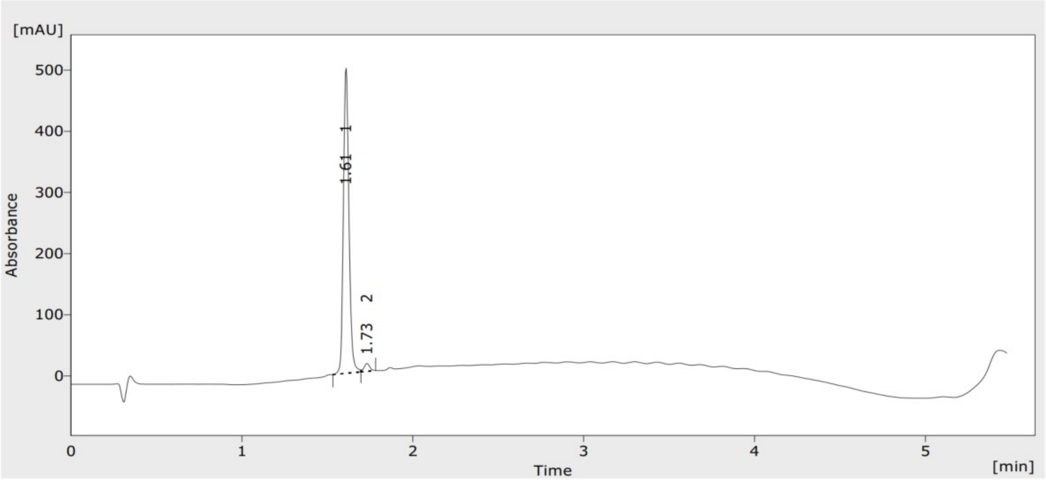
[mAU]
500
400
300
200
100
0
Absorbance
1.61 1
1.73 2
0
1
2
3
4
5
Time
[min]

4

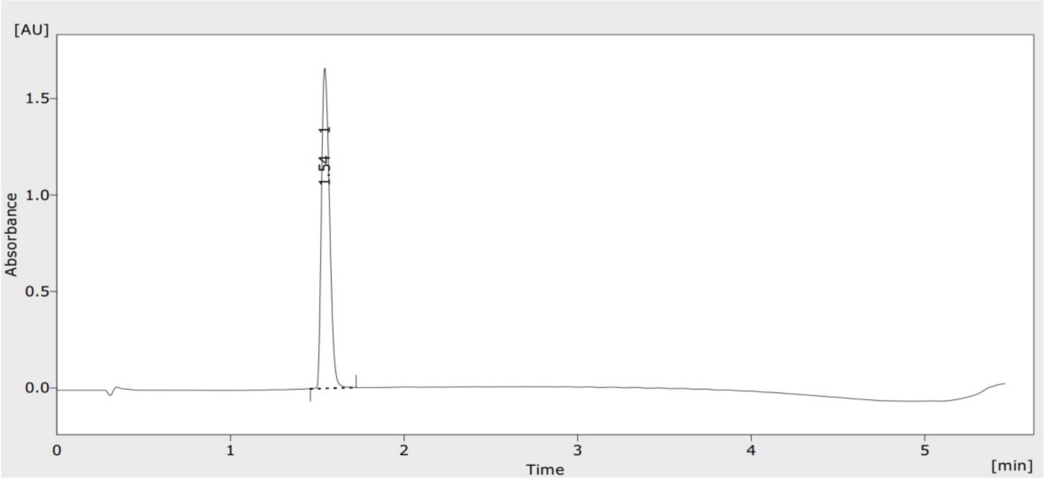
[AU]
1.5
1.0
0.5
0.0
Absorbance
1.54 1
0
1
2
3
4
5
Time
[min]

5

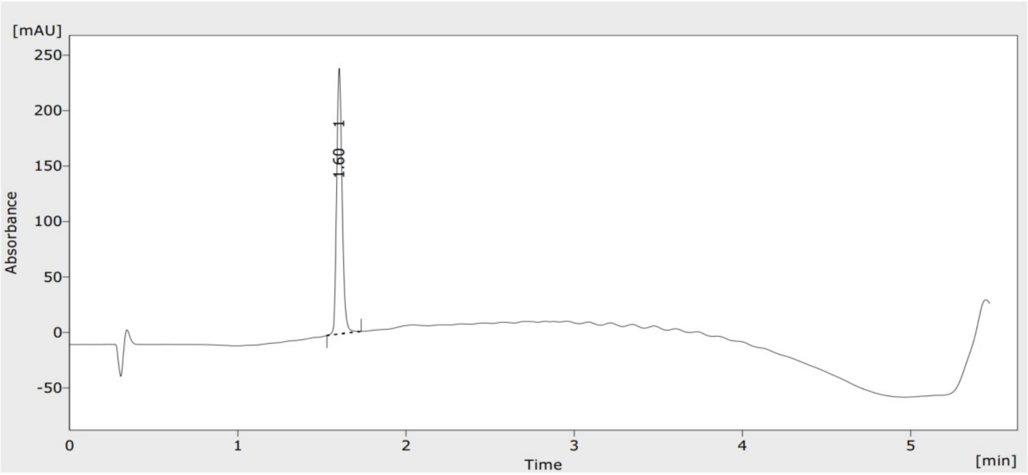

[mAU]
250
200
150
100
50
0
-50
Absorbance
1.60 1
0
1
2
3
4
5
Time
[min]

6

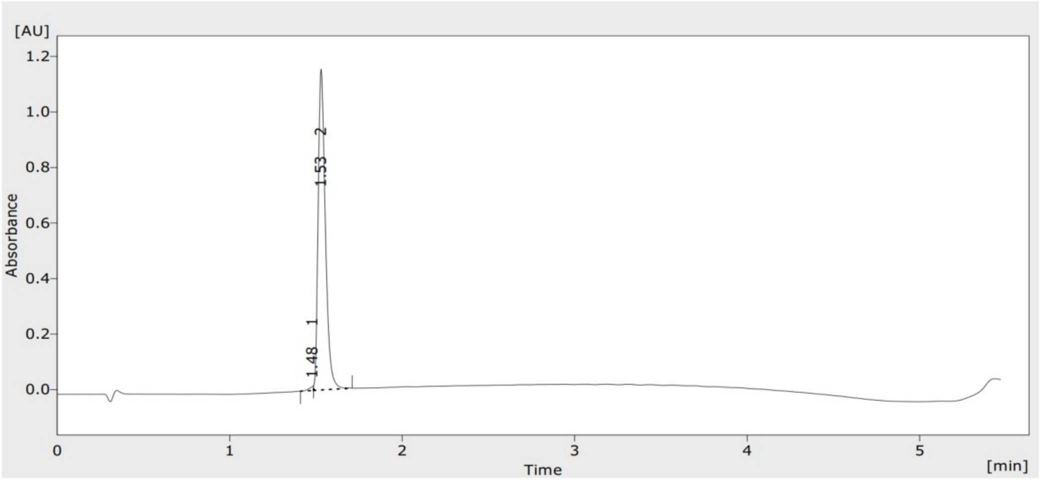

[AU]
1.2
1.0
0.8
0.6
0.4
0.2
0.0
Absorbance
1.53 2
1.48 1
0
1
2
3
4
5
Time
[min]

7

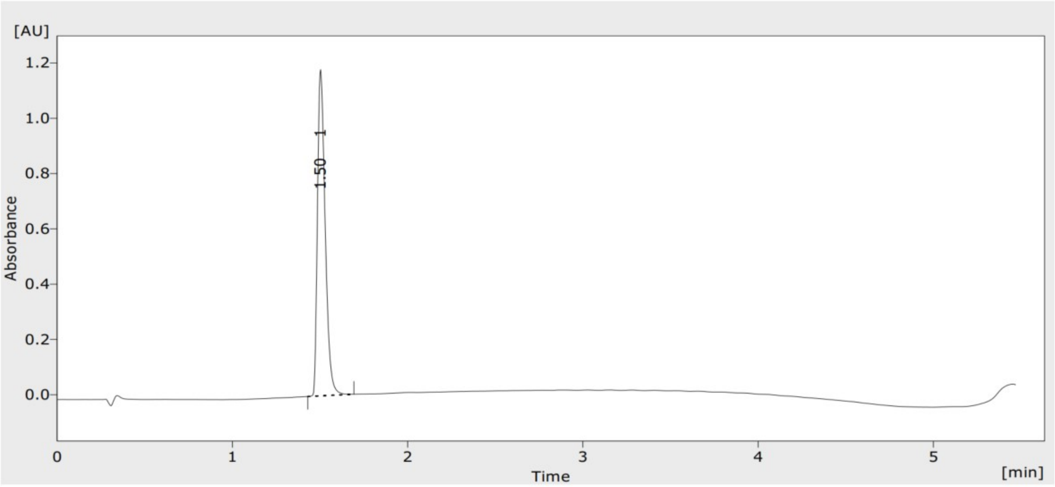

[AU]
1.2
1.0
0.8
0.6
0.4
0.2
0.0
Absorbance
1.50 1
0
1
2
3
4
5
Time
[min]

8

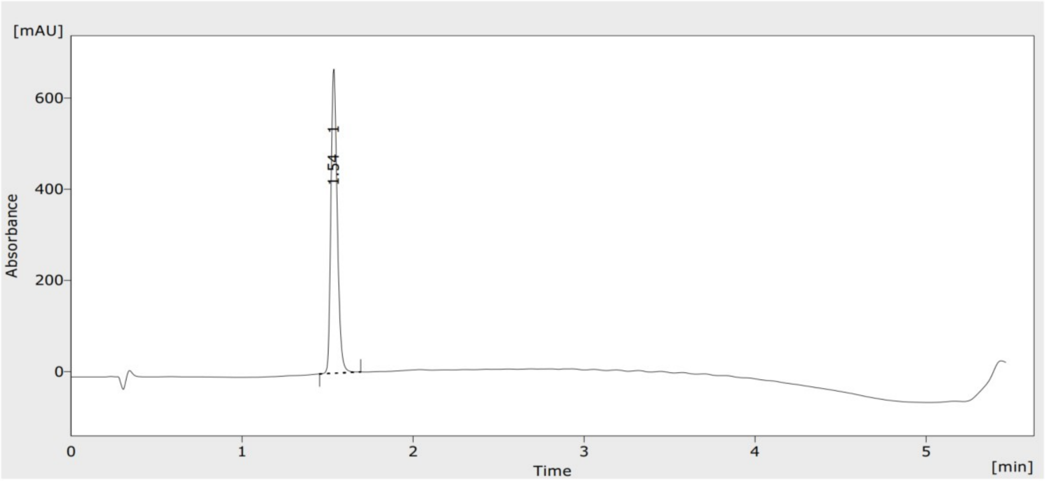
[mAU]
600
400
200
0
Absorbance
1.54 1
0
1
2
3
4
5
Time
[min]

9

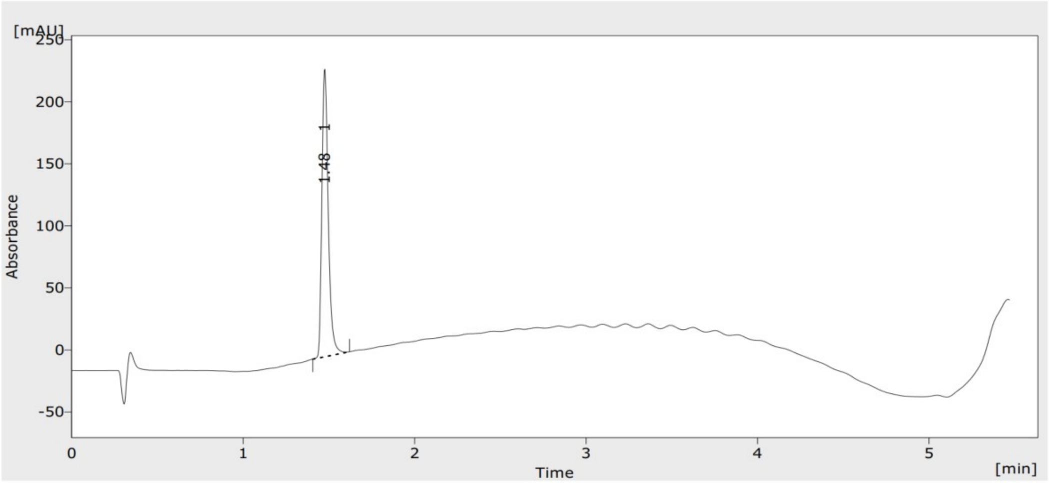
[mAU]
250
200
150
100
50
0
-50
Absorbance
1.48 1
0
1
2
3
4
5
Time
[min]

10

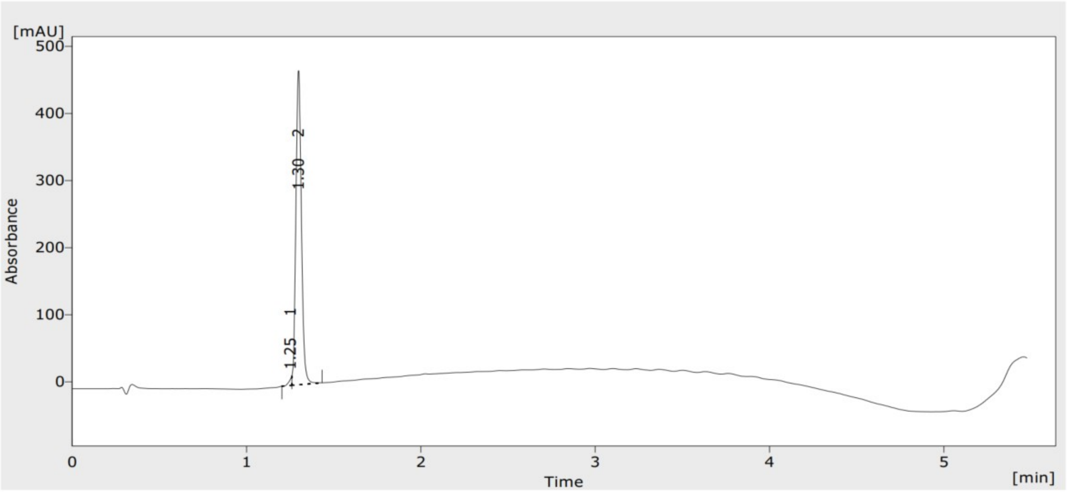
[mAU]
500
400
300
200
100
0
Absorbance
1.30 2
1.25 1
0
1
2
3
4
5
Time
[min]

11

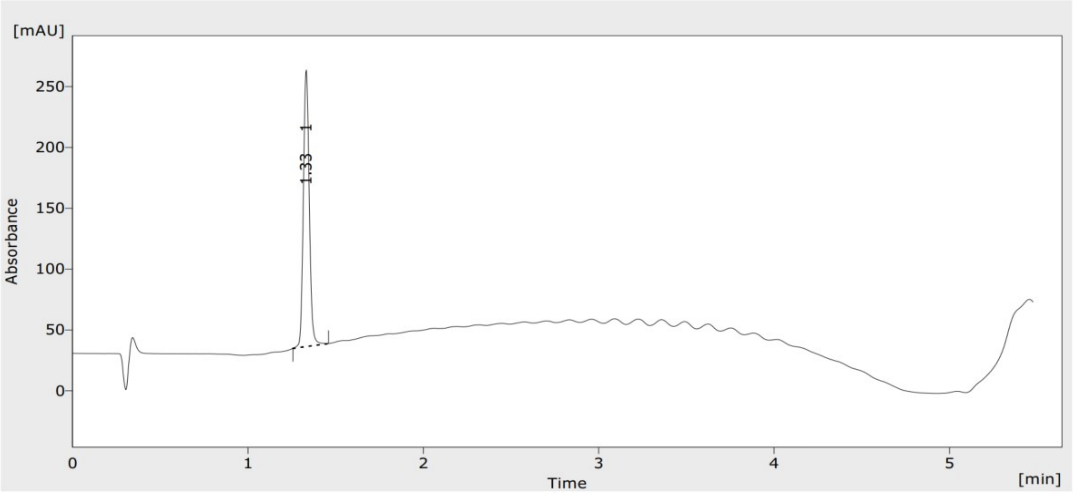
[mAU]
250
200
150
100
50
0
Absorbance
1.33 1
0
1
2
3
4
5
Time
[min]

12

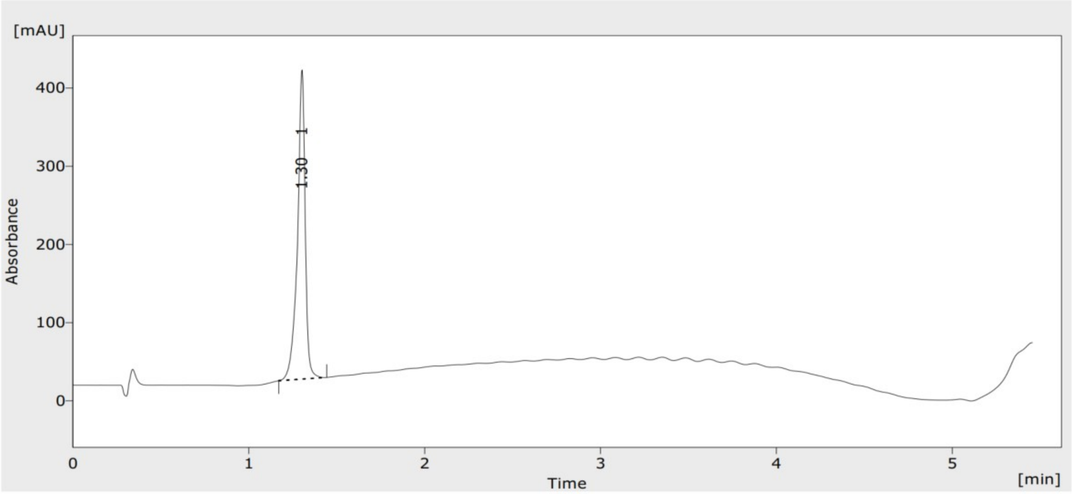
[mAU]
400
300
200
100
0
Absorbance
1.30 1
0
1
2
3
4
5
Time
[min]

13

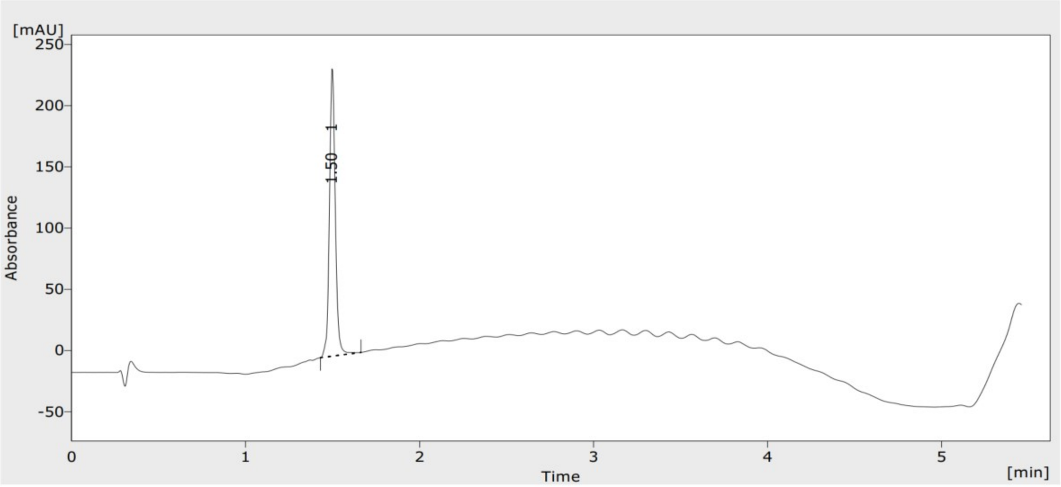
[mAU]
250
200
150
100
50
0
-50
Absorbance
1.50 1
0
1
2
3
4
5
Time
[min]

14

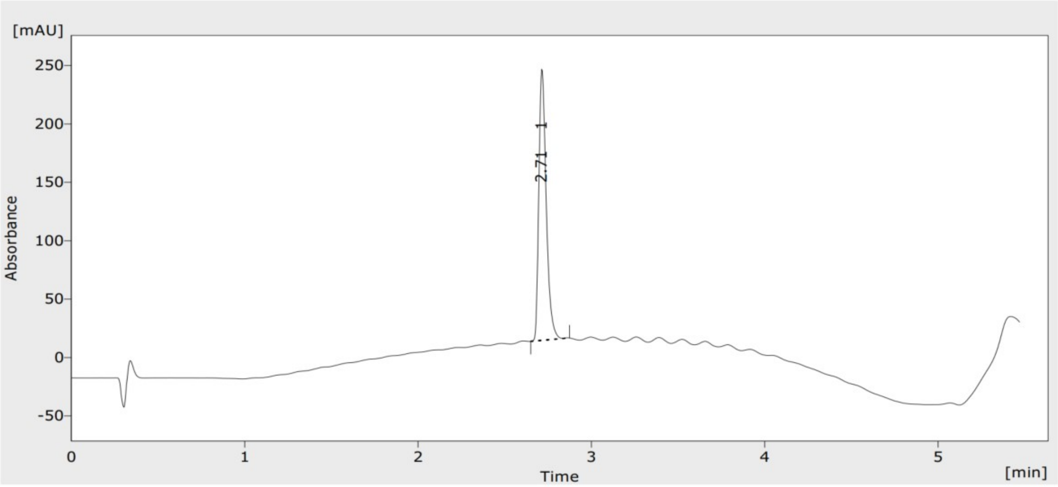

Tat-CT10

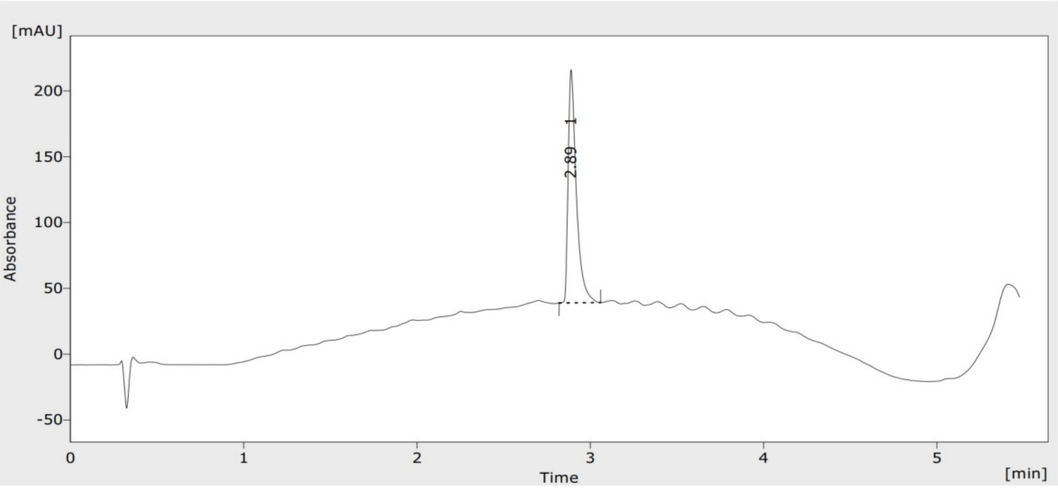

TAT-Gap19

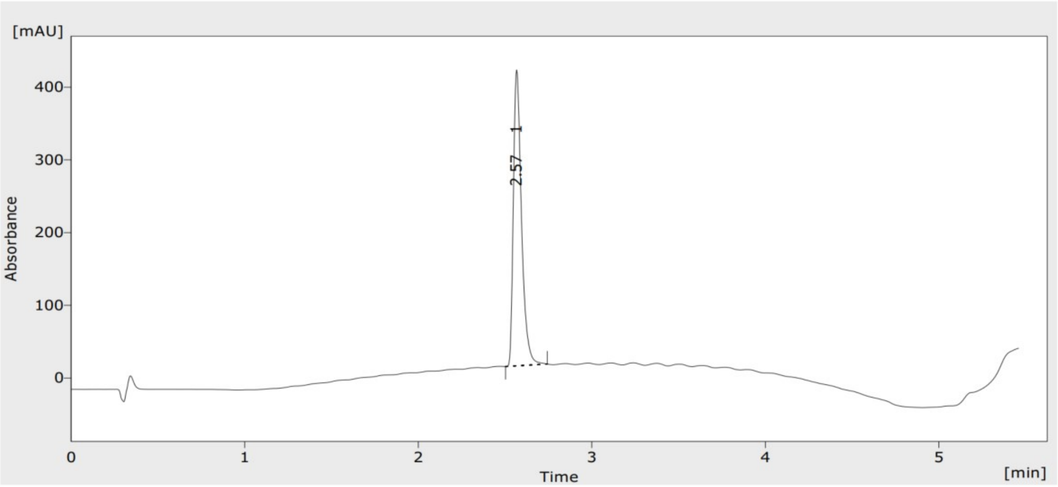

15

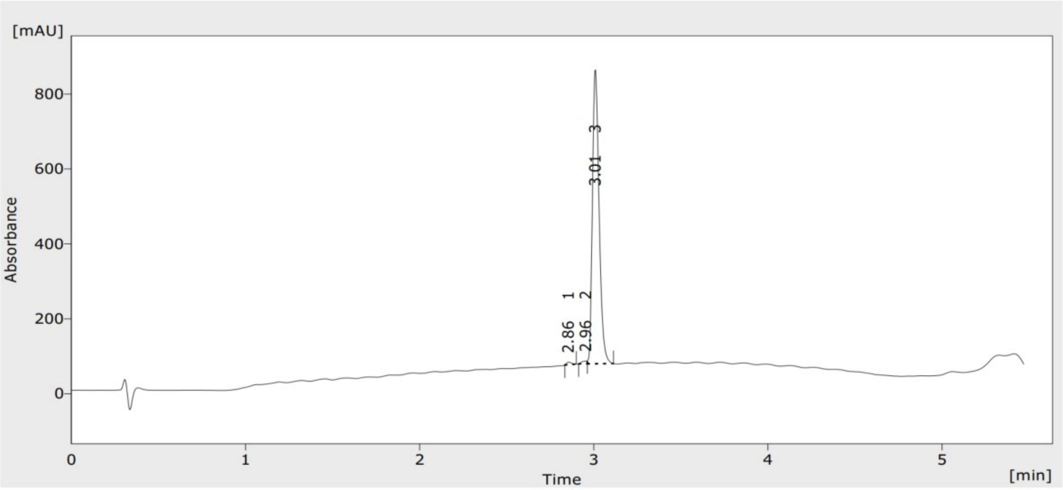
[mAU]
800
600
400
200
0
Absorbance
3.01 3
2.86 1
2.96 2
0
1
2
3
4
5
Time
[min]

16

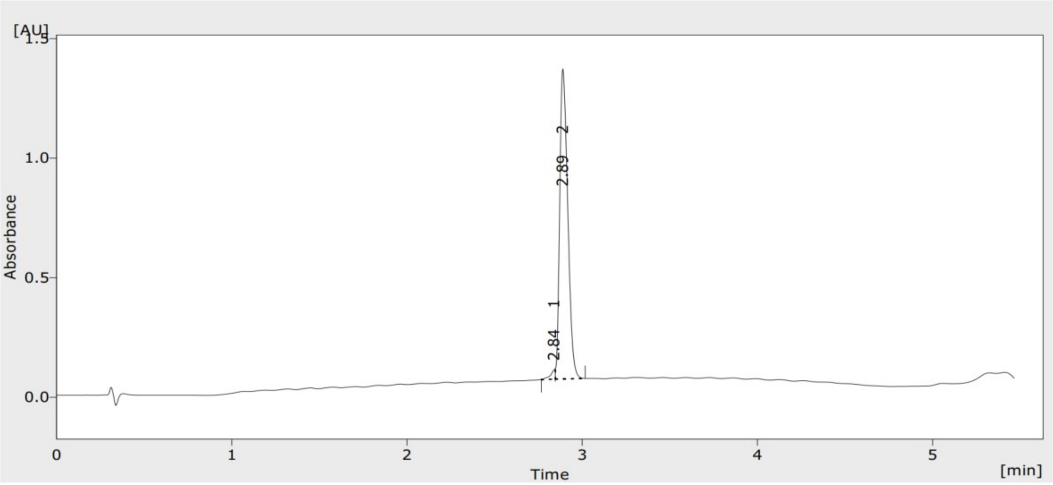
[AU]
1.5
1.0
0.5
0.0
Absorbance
2.89 2
2.84 1
0
1
2
3
4
5
Time
[min]

17

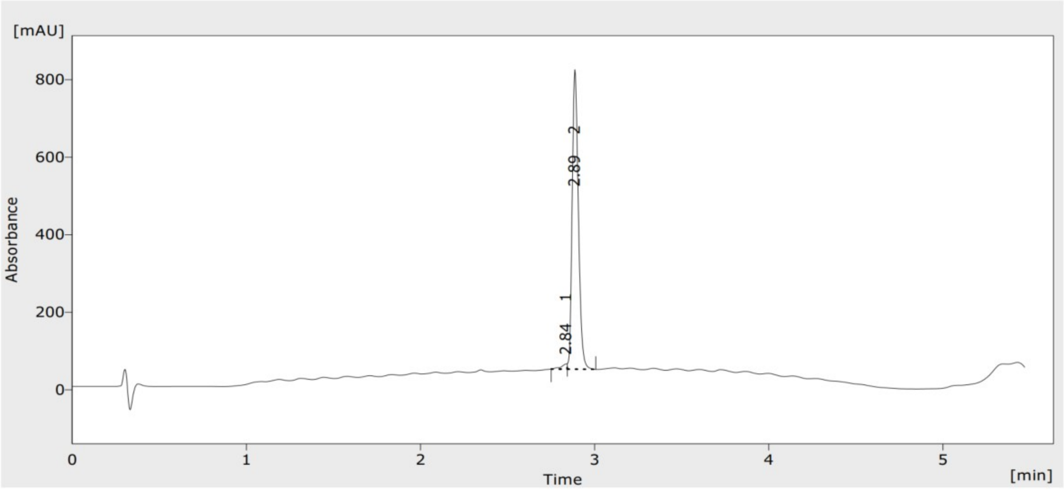
[mAU]
800
600
400
200
0
Absorbance
2.89 2
2.84 1
0
1
2
3
4
5
Time
[min]

18

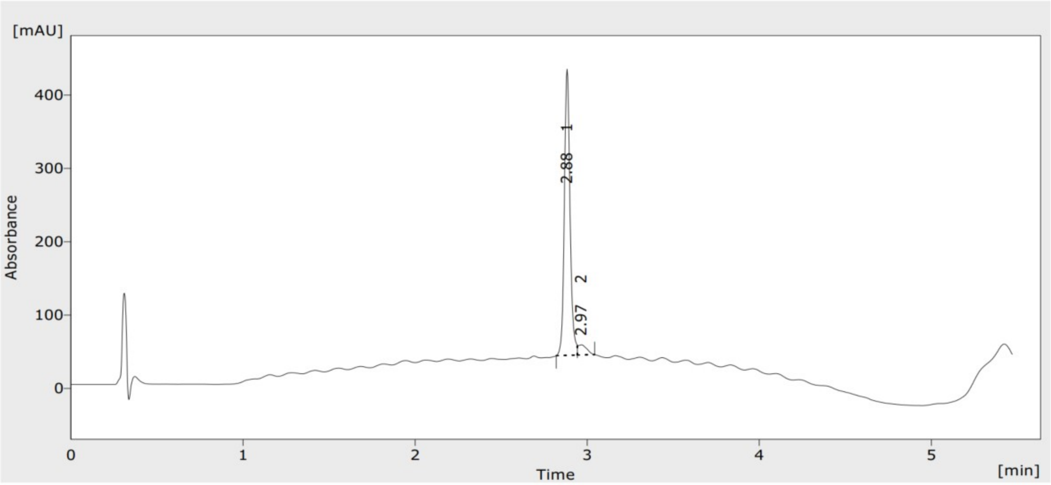
[mAU]
400
300
200
100
0
Absorbance
1
2.88
2
2.97
0
1
2
3
4
5
Time
[min]

19

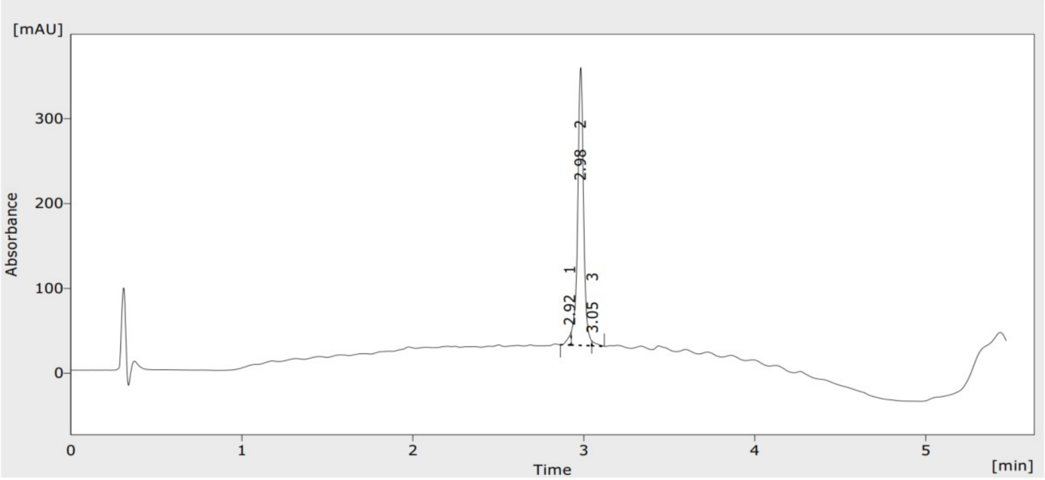
[mAU]
300
200
100
0
Absorbance
2
2.98
1
2.92
3
3.05
0
1
2
3
4
5
Time
[min]

20

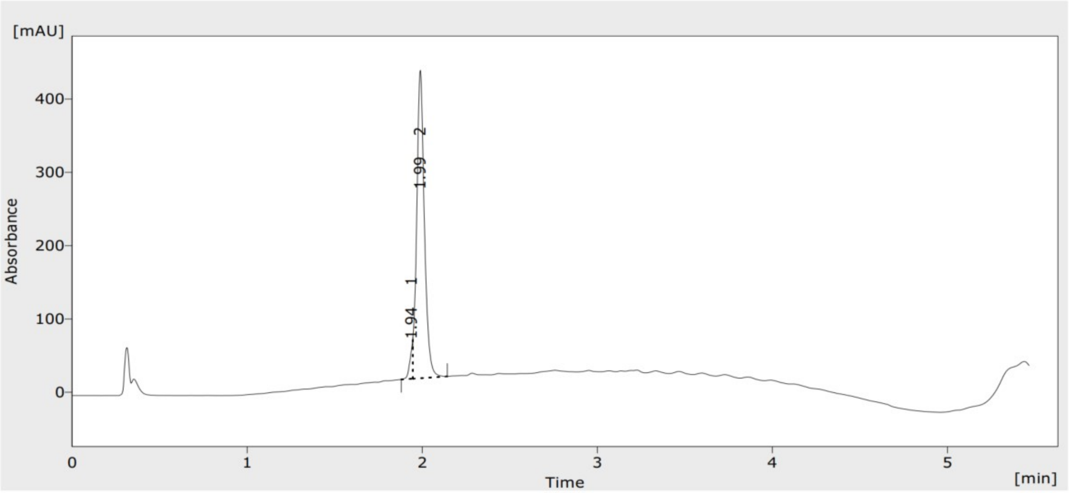
[mAU]
400
300
200
100
0
Absorbance
2
1.99
1
1.94
0
1
2
3
4
5
Time
[min]

21

3b. Characterization

Table S6. *a)[M+H]+; (b) as determined by RP-HPLC; (c) [M+2H]++ . Azp = (4S)-4-azidoproline, Pra = propargylglycine, Azk = azidolysine, Palm = palmitic acid, Ahx = 6-aminohexanoic acid. Residues between squares? are part of the peptide macrocycle.*

Peptide	Sequence	MW (TFA salt)	HRMS calculated [a]	HRMS found [a]	Rt [b] (min)	Yield (%)	Purity (%)	Formula
**αCT1**	H-Arg-Gln-Pro-Lys-Ile-Trp-Phe-Pro-Asn-Arg-Arg-Lys-Pro-Trp-Lys-Lys-Arg-Pro-Arg-Pro-Asp-Asp-Leu-Glu-Ile-OH	4398.09	1629.4277[(c)]	1629.4271[(c)]	1.78	17	95	C150H238N48O34
**CT10**	H-Ser-Arg-Pro-Arg-Pro-Asp-Asp-Leu-Glu-Ile-OH	1539.37	1197.6228	1197.6255	1.58	55	>99	C50H84N16O18
**Gap19**	H-Lys-Gln-Glu-Ile-Lys-Lys-Phe-Lys-OH	1731.57	1161.7360	1161.7383	1.52	49	99	C55H96N14O13
**TAT-Gap19**	H-Tyr-Gly-Arg-Lys-Lys-Arg-Arg-Gln-Arg-Arg-Arg-Lys-Gln-Ile-Glu-Ile-Lys-Lys-Phe-Lys-OH	4185.60	1351.8419 [(c)]	1351.8451 [(c)]	1.56	34	>99	C119H212N46O26
**Gap27**	H-Ser-Arg-Pro-Thr-Glu-Lys-Thr-Ile-Phe-Ile-Ile-OH	1646.63	1304.7578	1304.7594	2.03	49	>99	C49H85N13O12
**1 (SBL-CX43-106)**	Ac-Ser-Arg-Pro-Arg-Pro-Asp-Asp-Leu-Glu-Ile-OH	1467.38	1239.6333	1239.6327	1.62	77	>99	C52H86N16O19
**2 (SBL-CX43-107)**	H-Ser-Arg-Pro-Arg-Pro-Asp-Asp-Leu-Glu-Ile-NH_2	1538.37	1196.6388	1196.6404	1.47	16	99	C50H85N17O17
**3 (SBL-CX43-108)**	H-Ser-Ala-Pro-Arg-Pro-Asp-Asp-Leu-Glu-Ile-OH	1340.25	1112.5588	1112.5626	1.59	22	99	C47H77N13O18
**4 (SBL-CX43-109)**	H-Ser-Arg-Pro-Ala-Pro-Asp-Asp-Leu-Glu-Ile-OH	1340.25	1112.5588	1112.5614	1.61	44	98	C47H77N13O18
**5 (SBL-CX43-110)**	H-Ser-Arg-Pro-Arg-Pro-Ala-Asp-Leu-Glu-Ile-OH	1495.38	1153.6329	1153.6359	1.54	23	>99	C49H84N16O16
**6 (SBL-CX43-111)**	H-Ser-Arg-Pro-Arg-Pro-Asp-Ala-Leu-Glu-Ile-OH	1495.38	1153.6329	1153.6375	1.60	42	>99	C49H84N16O16
**7 (SBL-CX43-112)**	H-Ser-Arg-Pro-Arg-Pro-Asp-Asp-Leu-Ala-Ile-OH	1481.35	1139.6173	1139.6201	1.53	13	99	C48H82N16O16
**8 (SBL-CX43-113)**	H-Ser-Arg-Ala-Arg-Pro-Asp-Asp-Leu-Glu-Ile-OH	1513.35	1171.6072	1171.6097	1.50	20	99	C48H82N16O18
**9 (SBL-CX43-114)**	H-Ser-Arg-Pro-Arg-Ala-Asp-Asp-Leu-Glu-Ile-OH	1513.35	1171.6072	1171.6073	1.54	48	>99	C48H82N16O18
**10 (SBL-CX43-147)**	H-Ser-Arg-Ala-Arg-Ala-Asp-Asp-Leu-Glu-Ile-OH	1487.31	1145.5914	1145.5938	1.48	19	>99	C46H80N16O18
**11 (SBL-CX43-125)**	Ac-Ser-Arg-Pro-Arg-**[Azp**-Asp-Asp-Leu-Glu-**Pra]**-NH_2	1489.37	1261.6038	1261.6045	1.30	14	99	C51H80N20O18
**12 (SBL-CX43-138)**	Ac-Ser-Arg-Pro-Arg-**c[Pra**-Asp-Asp-Leu-Glu-**Azk]**-NH_2	1505.41	1277.6351	1277.6340	1.33	9	>99	C52H84N20O18
**13 (SBL-CX43-139)**	Ac-Ser-Arg-Pro-Arg-**c[Azp**-Asp-Asp-**Pra]**-Glu-Ile-NH_2	1489.37	1261.6038	1261.6038	1.30	9	>99	C51H80N20O18
**14 (SBL-CX43-140)**	Ac-**c[Pra**-Arg-Pro-Arg-**Azk]**-Asp-Asp-Leu-Glu-Ile-NH_2	1531.49	1303.6871	1303.6846	1.50	4	95	C55H90N20O17
**Palm-CT10 (SBL-CX43-135)**	Palm-Ser-Arg-Pro-Arg-Pro-Asp-Asp-Leu-Glu-Ile-OH	1663.78	1435.8524	1435.8545	2.71	26	99	C66H114N16O19
**Palm-Gap19 (SBL-CX43-137)**	Palm-Lys-Gln-Ile-Glu-Ile-Lys-Lys-Phe-Lys-OH	1855.97	1399.9656	1399.9698	2.89	31	>99	C71H126N14O14
**15 (SBL-CX43-152)**	Palm-εLys-PEG-Ser-Arg-Pro-Arg-Pro-Asp-Asp-Leu-Glu-Ile-OH	2051.13	1709.0212	1709.0217	2.57	6	98	C78H137N19O23
**16 (SBL-CX43-153)**	Palm-εLys-Ser-Arg-Pro-Arg-Pro-Asp-Asp-Leu-Glu-Ile-OH	1905.98	1563.9474	1563.9469	3.01	11	98	C72H126N18O20
**17 (SBL-CX43-154)**	Palm-Lys-Ser-Arg-Pro-Arg-Pro-Asp-Asp-Leu-Glu-Ile-OH	1905.98	1563.9474	1563.9492	2.89	24	98	C72H126N18O20
**18 (SBL-CX43-157)**	Palm-εLys-Ser-Arg-Pro-Arg-**c[Azp**-Asp-Asp-Leu-Glu-**Pra]**-NH_2	1927.95	1585.9178	1585.9183	2.89	4	98	C71H120N22O19
**19 (SBL-CX43-158)**	Palm-εLys-Ser-Arg-Pro-Arg-**c[Azp**-Asp-Asp-Leu-Glu-**Pra]**-Ahx-Lys(Fluorescein)-Ahx-Cys-Arg-Pro-Pro-Arg-NH_2	3476.57	1454.2767 [c]	1454.2802[c]	2.88	2	95	C135H207N37O33S
**20 (SBL-CX43-159)**	Palm-εLys-Ser-Arg-Pro-Arg-**c[Azp**-Asp-Asp-Leu-Glu-**Pra]**-Ahx-Lys(Fluorescein)-NH_2	2525.61	1093.0762 [c]	1093.0773 [c]	2.98	2	96	C104H153N25O27
**21 (SBL-CX43-160)**	Ac-Ser-Arg-Pro-Arg-**c[Azp**-Asp-Asp-Leu-Glu-**Pra]**-Ahx-Lys(Fluorescein)-Ahx-Cys-Arg-Pro-Pro-Arg-NH_2	3037.99	1292.1196 [c]	1292.1194 [c]	1.99	1	95	C115H167N35O32S
